# Turn-off mode fluorescent norbornadiene-based photoswitches†
†Electronic supplementary information (ESI) available: Synthetic procedures, ^1^H, ^13^C and COSY-NMR spectra, UV-Vis absorption, kinetics and Arrhenius analysis. Photoisomerization quantum yield, fluorescence lifetime measurements and TDDFT calculations data. See DOI: 10.1039/c8cp04329a


**DOI:** 10.1039/c8cp04329a

**Published:** 2018-08-16

**Authors:** Behabitu Ergette Tebikachew, Fredrik Edhborg, Nina Kann, Bo Albinsson, Kasper Moth-Poulsen

**Affiliations:** a Department of Chemistry and Chemical Engineering , Chalmers University of Technology , SE-41296 Gothenburg , Sweden . Email: balb@chalmers.se ; Email: mkasper@chalmers.se

## Abstract

Single-molecule fluorescence emission of certain positive photochromic systems such as diarylethenes have been exploited for biological imaging and optical memory storage applications. However, there is a lack of understanding if negative photochromic systems can be used for such type of applications. Hence, to explore the potential of negative photochromic molecules for possible optical memory storage applications, we have here synthesized and studied a series of four norbornadiene–quadricyclane (NBD–QC) photoswitching molecules. These molecules feature either linearly conjugated or cross-conjugated pi-electron systems. Upon photoisomerization, the UV-vis absorption spectra of the molecules revealed a strong blue shift in the QC-form, with a photoisomerization quantum yield close to 80% for the cross-conjugated systems. In contrast, a strong intrinsic emission (up to *Φ*_f_ = 49%) for the linearly conjugated compounds in the NBD form was observed. Upon light-induced isomerization, the emission was completely turned off in the QC-form in all the compounds studied. Further, the robustness of the system was evaluated by performing several switching cycles. Under nitrogen, the emission can be turned off and recovered with almost no loss of emission. We also show that the QC-form can be photochemically triggered to convert back to the NBD-form using a low energy UV light (340 nm), allowing an all optical conversion to both species. The demonstrated properties can make the NBD–QC system attractive for potential applications such as optical memory storage devices.

## Abstract 2

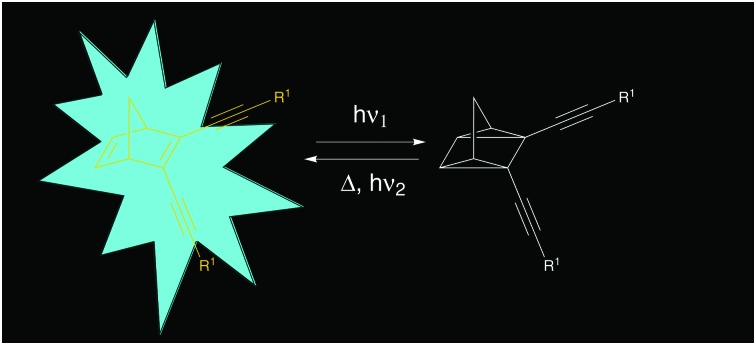
To explore the potential of negative photochromic molecules for possible optical memory storage applications, we have here synthesized and studied a series of four norbornadiene–quadricyclane (NBD–QC) photoswitching molecules.

## Introduction

The emission of light from certain photoresponsive materials upon photoexcitation has intrigued scientists in the past and led to a variety of applications.^1^,^2^ More recently, however, the ability to modulate the emission of the emitting species has drawn significant attention, as it lends itself for applications ranging from biological imaging to ultra-high density optical memories.^4^ In this context, photochromic molecules present an attractive opportunity since they can be tailored towards a specific application.

Photochromic molecules are molecules that change their colour reversibly upon irradiation with light. This colour change arises from a change in the structural and stereoelectronic arrangement in the molecule. Besides the colour change, a change in absorption,^5^ electrical conductance^4^,^6^ and dipole moment^7^,^8^ of the photochromic molecule can be manifested. Some photochromic molecules are intrinsically fluorescent in one form and non-fluorescent in the other isomer. Another way to impart fluorescence properties to a photochromic molecule is by attaching a fluorophore.^9^–^11^ In the latter case, the absorption of one of the isomers of the photochromic unit needs to be matched to the emission of the fluorophore so that efficient quenching occurs. Upon light-triggered photoisomerization, the fluorescence can be switched back on. This light induced modulation of the emissive properties of a photochromic molecule between the fluorescent and the non-fluorescent form has been successfully used in high-resolution fluorescence imaging techniques^12^–^15^ as well as ultrahigh density optical memories.^16^–^18^ For a fluorescent photochromic molecule to be used for such types of applications, certain requirements need to be met. Two of the most critical ones are fatigue resistance and high fluorescence quantum yield.^4^,^5^

**Scheme 1:**
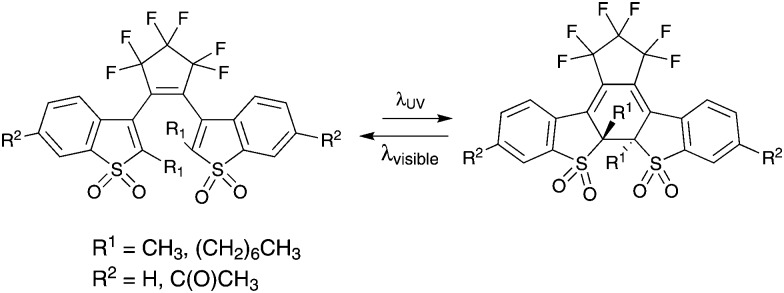
Examples of fluorescent DAE-based molecules.^22^

Various fluorescent photochromic molecules have been reported in the literature,^4^,^7^,^19^–^23^ where the most prominent so far has been the diarylethene (DAE)-based systems. Since most DAEs are not intrinsically fluorescent, a fluorescent unit such as anthracene,^24^ organic dyes^25^ or even nanoparticles^9^ have been attached to the DAE photochromic unit. Some DAE derivatives, *e.g.* benzothiophenes, are fluorescent in themselves; however, they suffer from poor fluorescence quantum yields. Recently, certain highly fluorescent DAE derivatives with a fluorescent quantum yield close to 90% were prepared by oxidizing the sulphur on the thiophene unit of the DAE to a sulphur-*S,S*-dioxide (Scheme 1).^26^ However, the photostability of these dioxides is reported to be lower than the unoxidized counterparts.^23^

**Scheme 2:**
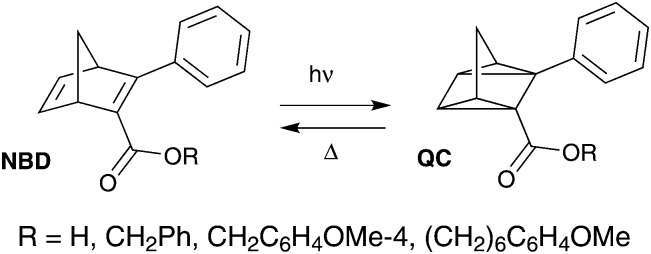
Examples of fluorescent NBD–QC systems.^37^

Another photochromic molecule that has seen a renewed interest recently due to possible applications in solar energy storage^27^–^30^ and data storage^3^ is the norbornadiene–quadricyclane (NBD–QC) system (Scheme 2). The NBD–QC pair is a T-type^5^ negative photochrome, where the unsaturated diene, *i.e.* the NBD-form, converts to the saturated QC-form upon photoirradiation. When desired, the QC-form can be triggered to relax back to the NBD-form thermally,^27^ electrochemically,^30^ by catalytic activation^31^ or even using light.^32^–^34^ This class of photochromic molecules has seen a recent rise in interest, since a relatively low energy photon is used to initiate the photoisomerization of the parent molecule to its isomer compared to positive photochromes.^35^ This is particularly attractive for biological applications as it is less damaging to cells and tissues.

There are few reported examples of fluorescent NBD–QC photochromic systems. Maafi *et al.* described one of the earliest examples of the fluorescence properties of norbornadiene derivatives containing an ester unit (Scheme 2).^36^ Interestingly, upon photoisomerization to its QC-form, the emission intensity increased. The lower emission in the NBD-form was attributed to intramolecular self-quenching of the phenyl ester fluorophore emission by the NBD unit. A similar behaviour was observed in a polyphenylene polymer containing a pendant norbornadiene moiety.^37^ Babudri *et al.* also reported a conjugated polymer containing an NBD backbone structure with a fluorescent quantum yield of 24%.^38^ However, long exposure to UV irradiation resulted in a significant decrease in the fluorescence quantum yield.

In the synthesis of various 2,3-disubstituted norbornadiene–quadricyclane (NBD–QC) photochromic systems, we have observed fluorescence in certain NBD-derivatives.^3^ Here, we report unprecedented emission properties for this class of photochromic systems, featuring a high fluorescence quantum yield in the NBD-form, a virtually non-emissive QC-form and excellent fatigue resistance. Furthermore, we demonstrate fluorescence modulation through multiple emission switching cycles by photoisomerization from NBD to QC and thermally activated back-conversion. Moreover, we also show the NBD to QC and QC to NBD switching, using relatively low energy UV (340 nm) sources, making this an entirely photoswitching system.

## Experimental details

### General procedures

All reagents and HPLC grade solvents were obtained from commercial sources and used as received. Dry solvents (toluene and tetrahydrofuran) were obtained from MBraun MB SPS-800 solvent purification system. All reactions were performed in oven-dried flasks under positive nitrogen pressure unless stated otherwise. Flash chromatography was conducted using a Biotage Isolera™ Spektra One flash chromatography system. Thin-layer chromatography (TLC) was performed on pre-coated aluminum plates with Merck Silica gel 60 F_254_ and the spots were visualized by using 254 and 365 nm handheld UV lamp. Infrared measurements were carried out using Perkin Elmer Frontier instruments provided with an ATR module.

Proton (^1^H), carbon (^13^C) and correlation spectroscopy (COSY) nuclear magnetic resonance (NMR) were recorded on an automated Agilent (Varian) MR 400 (400 MHz) spectrometer equipped with a “One-probe”. Chemical shifts are given in parts per million (ppm) downfield from tetramethylsilane referring to the residual proton signal (CHCl_3_: 7.26 ppm in ^1^H-NMR) and carbon signal (CDCl_3_: 77.0 ppm in ^13^C-NMR) in the NMR solvent. Data are presented as chemical shift, multiplicity (s = singlet, d = doublet, t = triplet, q = quartet, m = multiplet), coupling constants in Hertz (Hz) and integration. Absorption spectroscopy and kinetics experiments were carried out using a Cary 50, Cary 100 or Cary 4000 UV-vis spectrophotometer provided with a water heating system. Fluorescence emission spectra were recorded on a Spex Fluorolog 3 spectrofluorimeter from JY Horiba. Photoswitching experiments were performed using Thorlabs LED lamps 340 nm (M340L4), 365 nm (M365F1) and 405 nm (M405LP1). Photoisomerization quantum yields were measured according to a published literature method^39^ using a concentrated sample (absorbance > 2) at room temperature. The details are provided in Section V of the ESI.† High resolution mass (HRMS) was obtained using Agilent 1290 Infinity LC system tandem to an Agilent 6520 Accurate Mass Q-TOF LC/MS with an APCI source in a positive mode. Elemental analyses were performed at Mikrolab Kolbe.

The fluorescence quantum yield (*Φ*_F_) was measured using the procedure described in literature^40^ with Coumarin 102 in ethanol (*Φ*_F_ = 0.80) as reference. The concentration of the sample and reference sample was chosen so that both had the same absorption at the excitation wavelength. Excited state lifetime measurements were carried out using time correlated single photo counting (TCSPC) with a 377 nm excitation source from PicoQuant. An MCP-PMT detector was used, with 2048 channels and 10 000 counts in the top channel. The decay curves were fitted to mono- or bi-exponentials decays by deconvolution with the instrument response function. Vertical excitation energies and oscillator strengths were calculated by employing time dependent DFT (TDDFT) on DFT//B3LYP/6-31G** optimized structures.

### General procedure for the synthesis of the NBD compounds

In an oven-dried Schlenk flask (25 mL) provided with magnetic stirring bar, were added Pd(PPh_3_)_4_ (5 mol%), CuI (10 mol%), 2,3-dibromonorbornadiene (1.2 mmol, 1 eq.) and toluene (10 mL). The solution was purged with nitrogen for 15 minutes. Then, diisopropylamine (1 mL) and the alkyne (2.6 mmol, 2.1 eq.) were added, at which point the colour of the solution turned from yellow to dark red. The mixture was reacted until the starting materials were consumed as indicated by TLC. The solution was then diluted with dichloromethane and filtered through a short silica gel plug. The volatiles were then removed using a rotary evaporator. The crude product obtained was submitted to automated flash chromatography using an eluent gradient of 0–5% dichloromethane in hexane. The product was obtained after the solvent was removed *in vacuo*. (For the detailed synthetic procedure and characterization, see the ESI†).

## Results and discussion

### Synthesis

**Scheme 3:**
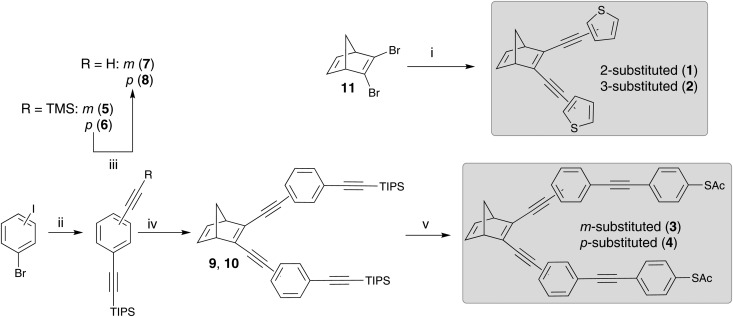
Synthetic routes to the norbornadiene derivatives **1–4** starting from bicyclo[2.2.1]hepta-2,5-diene (**11**): (i) 2-ethynyl thiophene (for **1**) or 3-ethynyl thiophene (for **2**), Pd(PPh_3_)Cl_2_, CuI, DIPA, 35 °C; (ii) (a) triisopropylsilyl acetylene, Pd(PPh_3_)_2_Cl_2_, CuI, DIPA, r.t., (b) trimethylsilyl acetylene, 60 °C, 12 h; (iii) K_2_CO_3_, MeOH:DCM, r.t.; (iv) **11**, Pd(PPh_3_)_2_Cl_2_, CuI, toluene, Et_3_N, 35 °C; (v) (a) TBAF, 0 °C to r.t., (b) 4-(iodophenyl)thioacetate, Pd(PPh_3_)_4_, CuI, toluene, 50 °C.

The synthesis of the NBD-derivatives, symmetrically substituted with a thiophenyl moiety as well as a thioacetate-terminated bis(ethynylphenylene), are shown in Scheme 3. The synthesis employed 2,3-dibromonorbornadiene (**11**) as the starting material, which in turn, was synthesized from bicyclo[2.2.1]hepta-2,5-diene (2,5-norbornadiene) according to a literature procedure.^3^ Compounds **1** and **2** were obtained in good yields (*ca.* 60%) *via* a Sonogashira reaction of 2- or 3-ethynylthiophene with **11**. The synthesis of **3** and **4** was achieved by first preparing **5** and **6** from the corresponding commercially available bromoiodobenzenes *via* a double Sonogashira reaction. Deprotection of the trimethylsilyl groups furnished the dialkynylated compounds **7** and **8** in excellent yields (>90%). Subsequent Sonogashira reaction of **7** and **8** with **11** led to the triisopropyl silyl (TIPS) protected intermediates **9** and **10**. Ultimately, deprotection of the TIPS groups using tributylammonium fluoride (TBAF), followed by a Sonogashira coupling with 4-iodophenyl thioacetate, yielded **3** and **4** in approximately 20 and 40% yield, respectively. It is worth noting that the synthesis of **4** has been described previously.^3^ In addition, all four compounds were characterized by elemental analysis, HRMS, IR and NMR (see ESI†).

### Steady state absorption spectra

**Fig. 1:**
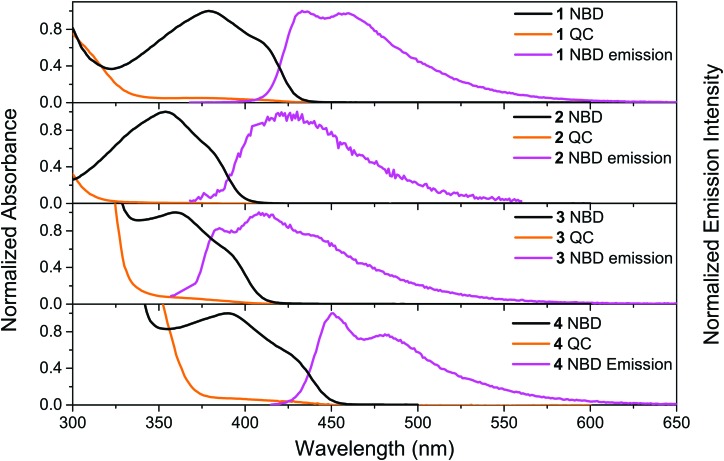
Normalized steady state absorption spectra (NBD- and QC-forms) of **1**, **2**, **3** and **4** and the corresponding emission spectra (*λ*_ex_ = 364, 362, 330 and 407 nm) in toluene.

The UV-vis absorption spectra of **1**–**4** in the NBD-form and QC-form were measured in toluene, see Fig. 1. For compounds **1-**NBD and **4**-NBD, that have the longest linearly conjugated systems, redshifted absorption spectra were observed in comparison to the respective cross-conjugated systems **2**-NBD and **3**-NBD. There is approximately a 50 nm difference between the onset of absorption (defined as log(*ε*) = 2) of the most redshifted spectrum of **4**-NBD compared to that of **2**-NBD, see Table 1. The absorption spectrum of **4**-NBD is not only redshifted, but also has the highest molar absorptivity of the four compounds studied (see ESI,† Fig. S13). The molar absorption coefficient (*ε*) (43 000 M^–1^ cm^–1^) of **4**-NBD at the first maximum (*λ*_max1_ = 391 nm) is about two times higher than the other three compounds and comparable to some of the strongest diarylethene absorbers reported in the literature.^26^

**Table 1:** Summarized photophysical properties of **1–4** in toluene

Compound	*λ*_max1_ ^*a*^ (nm) (*ε* (M^–1^ cm^–1^)); *λ*_onset_^*b*^ (nm)	*t*_1/2,25°C_ ^*c*^ (min)	*E*_a_ ^*d*^ (kJ mol^–1^)	*Φ*_NBD→QC_ ^*e*^	*Φ*_F_ ^*f*^	*τ*_F_ ^*g*^ (ns)	*k*_F_ ^*h*^ (s^–1^)	*k*_nr_ ^*i*^ (s^–1^)
**1**	381 (1.8 × 10^4^); 443	49	93.8	0.30	0.04	0.21	1.8 × 10^8^	4.6 × 10^9^
**2**	357 (1.8 × 10^4^); 415	201	103.7	0.54	0.002	0.02	1.0 × 10^8^	5.0 × 10^10^
**3**	364 (1.5 × 10^4^)	130	135.6	0.77	0.01	0.02	4.3 × 10^8^	5.0 × 10^10^
	301 (5.0 × 10^4^); 424							
**4**	391 (4.3 × 10^4^)	78.6	100.7	0.15	0.49	1.28	3.9 × 10^8^	3.9 × 10^8^
	332 (8.3 × 10^4^); 462							

^*a*^The first absorption maximum of NBD-forms.

^*b*^Absorption onset of the NBD-form is defined as the wavelength at which the molar absorptivity is log(*ε*) ≈ 2.

^*c*^Half-life of the thermal back reaction QC → NBD-forms at 25 °C.

^*d*^Activation energy for the thermal back reaction of QC → NBD-forms.

^*e*^Quantum yield for the photoisomerization of NBD-form to QC-form. The values given are the average of two measurements (see ESI Section IV).

^*f*^Fluorescence quantum yield of NBD-forms. The values given are the average of three measurements (see ESI Section V).

^*g*^Fluorescence lifetime of NBD-forms.

^*h*^Radiative rate constant.

^*i*^Nonradiative rate constant.

### Kinetics and photoisomerization quantum yields (*Φ*_NBD–QC_)

Irradiating compounds **1**-NBD and **4**-NBD with 405 nm light, and **2**-NBD and **3**-NBD with 365 nm light, converts the molecules **1–4** to their respective QC-forms with near quantitative conversion, as shown in Fig. 1. Moreover, no photostationary state was observed in any of the compounds studied, due to the strong blueshift in absorption onset of the QC-form relative to the NBD-form. Since the NBD–QC pair is a thermal photoswitch system, the thermal recovery of the QC-forms of **1–4** was also measured (see ESI,† Section III). The corresponding Arrhenius parameters and half-lives are presented in Table 1. All four compounds have relatively short half-lives (*ca.* 1 h to 3 h at 25 °C) as a result of the low average activation energy (*E*_a_) (*ca.* 100 kJ mol^–1^). In general, the linearly conjugated compounds **1**-QC and **4**-QC have a lower *E*_a_ than the cross-conjugated compounds **2**-QC and **3**-QC, hence they have shorter half-lives. The NBD–QC photoisomerization quantum yields (*Φ*_NBD→QC_) of **1–4** were measured using a Ferrioxalate actinometer^41^ (see ESI,† Section IV). The results are presented in Table 1. The cross-conjugated compounds **2**-NBD and **3**-NBD are found to have higher photoisomerization quantum yields in comparison to the corresponding linearly conjugated compounds **1**-NBD and **4**-NBD, respectively. In particular, **3**-NBD stands out with a photoisomerization quantum yield of around 80%.

### Steady state fluorescence spectra, fluorescence quantum yields and lifetimes

The emission spectra of **1–4** were recorded in toluene and the spectra are shown in Fig. 1. All four compounds show emission upon photoexcitation in the NBD-form. The fluorescence quantum yields of the four compounds in toluene were measured using Coumarin 102 as reference (*Φ*_F_ = 0.80) and the results are shown in Table 1. Out of the four compounds, **4**-NBD has the highest fluorescence quantum yield of 49%, followed by **1**-NBD of 4%. This can be compared to the much lower fluorescence quantum yield of 1% and 0.2% for the respective cross conjugated compounds **3**-NBD and **2**-NBD.

To fully understand the underlying cause for such a high variation in the fluorescence quantum yield, the excited state lifetimes of **1–4**-NBD were measured using time-correlated single photon counting (TCSPC). The fluorescence decay curves were fitted to a mono- or bi-exponential function, but only the major time constant with the highest amplitude was considered (for detailed information see ESI,† Section VI). The result of the measured lifetimes, *τ*_F_, and calculated radiative rate constants, *k*_F_, are summarized in Table 1. Previously, it has been reported that compounds with longer linear conjugation are considered to exhibit stronger fluorescence.^42^ As expected, the longest linearly conjugated compound **4**-NBD was found to have the highest fluorescence as well as a longer-lived excited state (1.28 ns), which is six times longer compared to compound **1**-NBD, and more than 50 times longer than the excited state lifetime of compound **2**-NBD and **3**-NBD. Moreover, since substituted NBDs have higher propensity to undergo photoisomerization to the QC-form on the singlet surface upon direct excitation,^43^ it is worth noting that the photoisomerization and fluorescence quantum yields of compounds **1–4** are complementary, similar to Stilbenes,^44^*i.e.* higher *Φ*_NBD→QC_ results in lower *Φ*_F_ and *vice versa*.

The result of the measured lifetimes, *τ*_F_, and calculated radiative (*k*_F_) and non-radiative rate constants (*k*_nr_) are presented in Table 1. The fluorescence rate constants, *k*_F_ = *Φ*_F_/*τ*_F_, of the related NBD-derivatives **1** and **2**, and **3** and **4** are nearly similar. However, the non-radiative rate constants (*k*_nr_) differ by an order of magnitude between the linearly conjugated compounds **4**-NBD and **1**-NBD. This difference is about two orders of magnitude between compound **4**-NBD and the cross-conjugated compounds **2**-NBD and **3**-NBD. Time dependent density functional theory (TDDFT) was used to calculate the excited state energies as well as the oscillator strength of transition from ground state to the first singlet excited state for compounds **1–4** in the NBD form, see ESI† for details. The calculated singlet excited state energies are consistent with the trend in the lower energy *λ*_max_ for the fully conjugated compounds compared to the respective cross-conjugated compounds, as observed in the absorption measurements. Also, the calculated oscillator strength shows that all four compounds have strong and allowed transition from ground state to the first singlet excited state. The oscillator strength is similar for compounds **1**-NBD and **2**-NBD, and slightly higher for **3**-NBD and **4**-NBD, which is in line with the radiative rate of fluorescence presented in Table 1. Generally, the results from emission measurements and TDDFT calculations show that the difference in fluorescence quantum yields of the four compounds are due to the large differences in the non-radiative decay mechanisms (Table 1).

Upon photoisomerization to the QC-form, the emission intensity for compounds **1–4** decreases to essentially zero, contrary to those reported by Maafi *et al.*^36^,^37^ A plausible explanations for this observed difference is that the emission, in the case of Maafi *et al.*, is due to the aromatic esters, and the fact that NBDs are known quenchers.^45^ Nevertheless, upon photoisomerization, NBDs are progressively converted to QCs, thus diminishing the quenching process. In our case, we did not observe an increase in emission. Hence, the fluorescence can be inferred to arise from the entire conjugated molecule. In addition, during the photoisomerization from NBD to QC, the conjugation becomes interrupted in compounds **1–4** at half-way, since the double bond becomes a single bond. This process turns off the emission in the QC-form, even for the longer conjugated molecules **3**-QC and **4**-QC. In fact, no emission was measured in the QC-form from any of the four compounds studied.

**Fig. 2:**
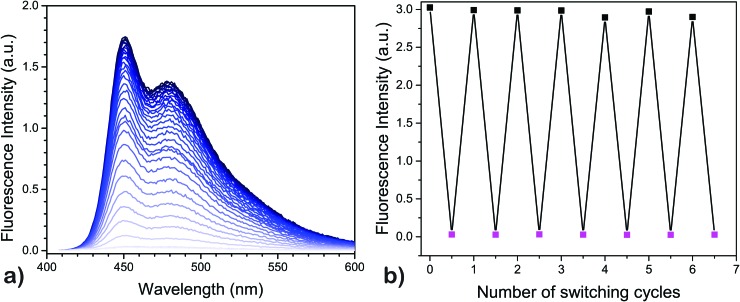
(a) Fluorescence recovery of **4** upon thermal relaxation of the QC-form to NBD-form at 50 °C in toluene, and (b) cyclability of the modulation of emissive property of **4** in toluene at 6 °C. The fluorescence switching ratio between the NBD-form and QC-form is about 100.

Once the QC-form is formed, it has several possible mechanistic pathways to revert back to the NBD-form,^30^–^32^ where one of the most common routes is by thermal activation.^27^ When the QC-form is heated, it relaxes back to the NBD-form. The thermal recovery of fluorescence, going from **4**-QC to the corresponding **4**-NBD, was recorded at 50 °C under nitrogen and the result is shown in Fig. 2a. A quantitative recovery of the NBD-form emission was observed at this temperature.

Once modulation of fluorescence was realized upon light-activated photoisomerization of the NBD-form form to the QC-form, the fluorescence fatigue resistance was tested by carrying out multiple photoisomerization-thermal recovery steps, using **4** as a model compound. The fluorescence measurements for the NBD-form and QC-form were carried out at 6 °C to slow down the thermal back-conversion of the QC-isomer since the half-life of **4**-QC at 25 °C is less than 2 h (Table 1, entry 4). However, at 6 °C, it is well over 8 h, as obtained by extrapolating the data, which was optimal to significantly reduce residual **4**-NBD emission for the **4**-QC isomer. In between the measurements, the cuvette was kept for 30 minutes at 50 °C in a heating chamber to thermally recover the NBD-form. About 7 cycles were measured with full recovery of fluorescence of **4**-NBD (Fig. 2b). The emission recovery pattern is found to follow a similar trend as the cyclability of photoisomerization of **4**.^3^ This means that under nitrogen, compound **4**-NBD has virtually no loss of emission. In air, some minor degradation (about 0.2% per cycle) was observed for compound **4**.^3^

**Fig. 3:**
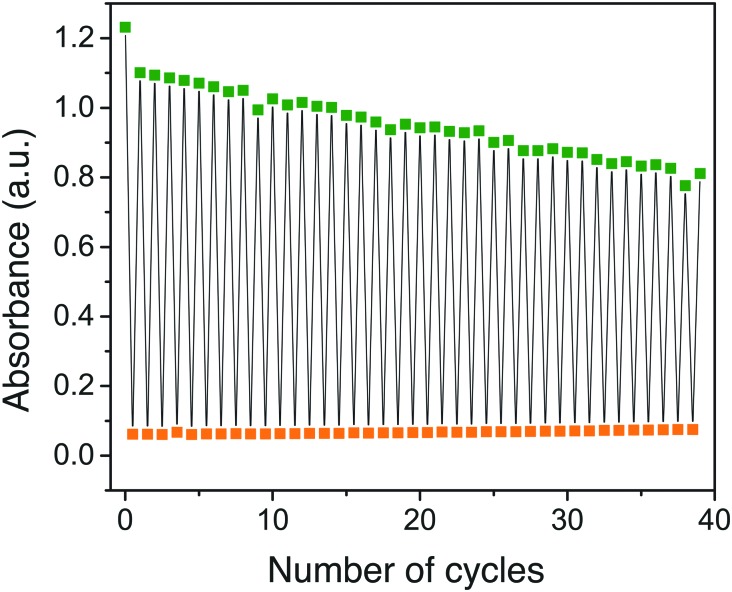
Dual light switching of compound **4**. Upon irradiation with a 405 nm LED lamp, the NBD form (green) converts to the QC form (orange) in about 60 s. Similarly, the QC form converts to the NBD form readily upon irradiation with a 340 nm LED lamp while change in absorbance was probed at 430 nm. The rate of degradation per cycle is faster than for the previously reported photo-thermal cycle.^3^

Furthermore, since heat-induced back-isomerization takes a few hours at 25 °C, we investigated light-induced back isomerization of **4**-QC to **4**-NBD using a 340 nm LED lamp (Fig. 3). Gratifyingly, the NBD-form was formed readily in less than a minute. This is an important feature that can be used to interconvert the two isomers in short period of time. Although light-induced QC to NBD back-isomerization has been reported previously,^32^–^34^ our system utilizes a relatively low energy near-UV light. This makes our system attractive for possible biological applications.

In summary, photoisomerization quantum yields of the linearly conjugated **1**-NBD and **4**-NBD have relatively large values compared to the cross conjugated forms **2**-NBD and **3**-NBD. In particular, the marked difference between **3**-NBD (*Φ*_NBD→QC_ = 77%) and **4**-NBD (*Φ*_NBD→QC_ = 15%) is worth noting. Moreover, compounds **1–4** are fluorescent in the NBD-form, with quantum yields ranging from <1% to 49% with **4**-NBD having the highest value. The photoisomerization and fluorescence quantum yields are found to be complementary. Furthermore, the effect of π-conjugation due to *meta*/*para*-arrangement of the bonds in the respective molecules was found to play a significant role, particularly for compounds **3**-NBD and **4**-NBD.

In contrast, none of the four compounds show any emission from the QC isomer. Emission spectra recorded from the samples after photoisomerization only shows a weak emission profile, which resembles the emission spectrum of the NBD-isomer. This emission is therefore assigned to residual NBD-form of the molecules that did not photoisomerize. From Fig. 1 it is clear that all four compounds have a wavelength region where the NBD isomer has a high absorptivity. However, the absorptivity of the respective QC isomer is essentially zero at *λ*_max1_. This means that all four compounds **1–4** in principle could be photoisomerized 100% from the NBD to the QC form without any photostationary states. The only limiting factor for complete photoisomerization is the thermally activated back-isomerization from QC to NBD. For compound **4**-QC, the half-life for the thermal relaxation ranges from about 80 minutes at 25 °C to less than 6 minutes at 50 °C. Hence, for short-term memory storage applications, compound **4** could be a promising photoswitch and its fluorescence emission, which can also be turned off to essentially zero emission by photoisomerization, can be used as a readout. Moreover, we have also demonstrated that it is possible to back-isomerize the QC-form to the NBD-form instantaneously using light, making it an entirely light-activated photoswitch. Thus, the remarkable stability and high fatigue resistance of compound **4** demonstrated through a cyclability test (Fig. 2b), particularly under nitrogen, makes it an appealing photochromic system for optical memory storage applications.

## Conclusions

We have synthesized and studied four fatigue resistant NBD/QC-based negative photochromic systems with conjugated and cross-conjugated electronic arrangements, and investigated their emission properties. The conjugated derivatives are found to be more emissive compared to the cross conjugated analogues. In particular, the linearly conjugated NBD-form of compound **4** is found to show a remarkable fluorescence quantum yield (49%). This is the highest value for an NBD-based photochromic system that has been reported so far. In contrast, the cross-conjugated analogues show a high photoisomerization quantum yield (about 80%). We have demonstrated multiple alternating photoactivation and thermal relaxations compound **4** to turn off and recover its emission in the NBD-form. Moreover, in addition to heat, low energy UV (340 nm) light was found to induce the back isomerization of QC-form to the NBD-form in less than a minute, making it a fast-responsive and all-light operated photoswitch system. In particular, compound **4**, with an all-light activated switching, remarkable fatigue resistance, and high emission, can potentially be used as a vital component in optical memory storages and imaging applications with a fluorescence readout.

## Conflicts of interest

There are no conflicts to declare.

## Supplementary Material

Supplementary information
