# Supplementary material for: Turn-off mode fluorescent norbornadiene-based photoswitches
Source: Phys Chem Chem Phys. 2018 Aug 16;20(36):23195–201. doi: 10.1039/c8cp04329a (PMC6146619; doi:10.1039/c8cp04329a)
Supplement: Supplementary file 1 [file CP-020-C8CP04329A-s001.pdf]

Electronic Supplementary Material (ESI) for Physical Chemistry Chemical Physics.
This journal is © the Owner Societies 2018

Turn-off mode fluorescent norbornadiene photoswitches

Behabitu Ergette Tebikachew, [a] Fredrik Edhborg, [a] Nina Kann[a] Bo Albinsson *[a] and Kasper Moth-Poulsen *[a]

Department of Chemistry and Chemical Engineering, Chalmers University of Technology, SE-41296 Gothenburg, Sweden.

E-mail: kasper.moth-poulsen@chalmers.se, balb@chalmers.se

Electronic Supplementary Information

Table of Contents

I. SYNTHETIC PROCEDURES 2

II. NUCLEAR MAGNETIC RESONANCE (NMR) DATA 8

III. ULTRAVIOLET-VISIBLE (UV-VIS) SPECTROPHOTOMETRIC STUDY 14

IV. PHOTOISOMERIZATION QUANTUM YIELD DETERMINATION USING FERRIOXALATE ACTINOMETRY 19

V. UV-VIS ABSORPTION AND EMISSION MEASUREMENTS 24

VI. BACK-ISOMERIZATION OF QC-FORM USING LIGHT 25

VII. REFERENCES 25

I. Synthetic procedures

Triisopropyl(3 (or 4)-((trimethylsilyl)ethynyl)phenyl)ethynyl silane (5 or 6):

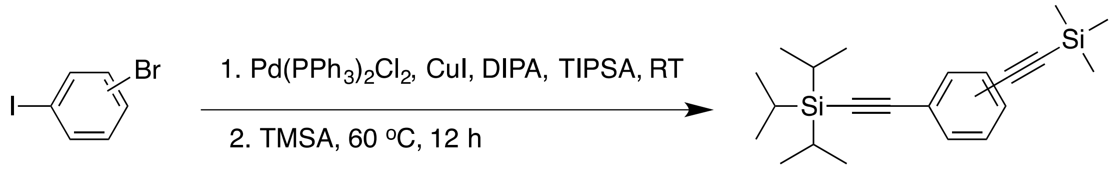

The synthesis of triisopropyl (3 (or 4)-((trimethylsilyl)ethynyl)phenyl)ethynyl silane (**5** or **6**) were carried out based on a modified literature procedure 1.[1] In a 250 mL two-necked round bottom flask, 3- or 4-bromoiodobenzene (23.5 mmol), $Pd(PPh_3)_2Cl_2$ (4 mol%), and CuI (4 mol%) were added, followed by diisopropylamine (100 mL). Then triisopropylsilyl acetylene (25 mmol) was added dropwise and the reaction was stirred at room temperature for 24 h. Then trimethylsilyl acetylene (25 mmol) was added dropwise and the reaction was stirred at 60 ^{o}C overnight. The solution was cooled and filtered through a short silica gel column with dichloromethane. The solution was washed with deionized water. The organic phase was collected and the solvent was removed *in vacuo*. The crude was then submitted to automated chromatography using hexane as the eluent at a flowrate of 50 mL/min to collect the product after removing the hexane *in vacuo*.

Meta-product (**5**): off-white liquid, 1.93 g (51%)

^{1}H NMR (400 MHz, $CDCl_3$) δ = 7.58 (td, *J* = 1.7, 1.7, 0.6 Hz, 1H), 7.40 (m, 2H), 7.23 (m, 1H), 1.12 (s, 21H), 0.25 (s, 9H).

^{13}C NMR (101 MHz, $CDCl_3$) δ = 135.49, 132.10, 131.81, 128.30, 123.88, 123.45, 106.19, 104.27, 94.99, 91.44, 18.81, 11.44, 0.07.

Para-product (**6**): light yellow oil, 2.68 g (71%)

^{1}H NMR (400 MHz, $CDCl_3$) δ = 7.39 (d, *J* = 0.4 Hz, 4H), 1.12 (d, *J* = 1.0 Hz, 21H), 0.25 (s, 9H).

^{13}C NMR (101 MHz, $CDCl_3$) δ = 131.95, 131.86, 123.71, 123.10, 106.72, 104.78, 96.29, 92.95, 18.81, 11.46, 0.07.

3 (or 4)-Ethynyl triisopropyl(phenylethynyl)silane (7 or 8)

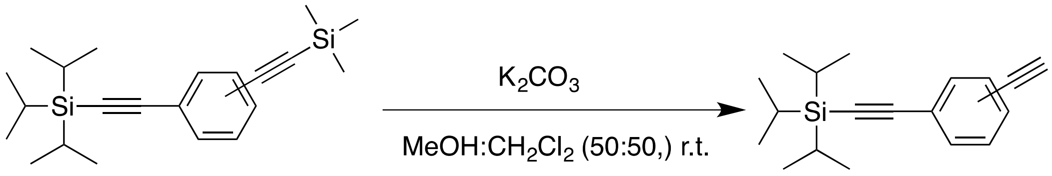

In a 250 mL round bottom flask provided with magnetic bar, compound **5** or **6** (7.5 mmol) was dissolved in dichloromethane (75 mL) and methanol (75 mL) and purged with nitrogen. Then potassium carbonate (2.2 eq.) was added and the solution was stirred under nitrogen at room temperature until the starting material was consumed, as indicated by TLC (hexane, 100%). The solid residue was filtered off and the solvent was removed *in vacuo*. Then the crude product was dissolved using dichloromethane, washed with deionized water and then with brine. The organic phase was separated, dried over anhydrous $MgSO_4$, filtered and the solvent was removed *in vacuo* to afford a crude product, which was further purified using automated chromatography with hexane as eluent at a flow rate of 50 mL/min. The product was collected after removing the hexane in vacuo.[1]

Deprotected *meta*-product (**7**): Off-white oil, 1.96 g (92%)

^1H NMR (400 MHz, $CDCl_3$) δ = 7.60 (td, *J* = 1.7, 1.7, 0.5 Hz, 1H), 7.43 (ddt, *J* = 9.9, 7.8, 1.4, 1.4 Hz, 2H), 7.27 (m, 1H), 3.08 (s, 1H), 1.13 (d, *J* = 0.8 Hz, 21H). *Note: the solvent* $CDCl_3$ *peaks overlapped with the aromatic proton signal.*

^{13}C NMR (101 MHz, $CDCl_3$) δ = 135.69, 132.40, 131.96, 128.43, 124.02, 122.44, 106.03, 91.73, 82.93, 77.85, 18.80, 11.43.

Deprotected *para*-product (**8**): light yellow oil, 1.92 g (90 %)

^1H NMR (400 MHz, $CDCl_3$) δ = 7.43 (s, 4H), 3.16 (s, 1H), 1.14 (q, *J* = 1.2, 1.2, 0.8 Hz, 21H).

^{13}C NMR (101 MHz, $CDCl_3$) δ = 132.04,* 124.13, 122.07, 106.54, 93.12, 83.41, 78.99, 18.80, 11.46. (*The four aromatic -CH- carbons have merged into one in the ^{13}C spectrum.)

***Meta*- and *para*-oligophenylethynyl NBD (9 or 10)**

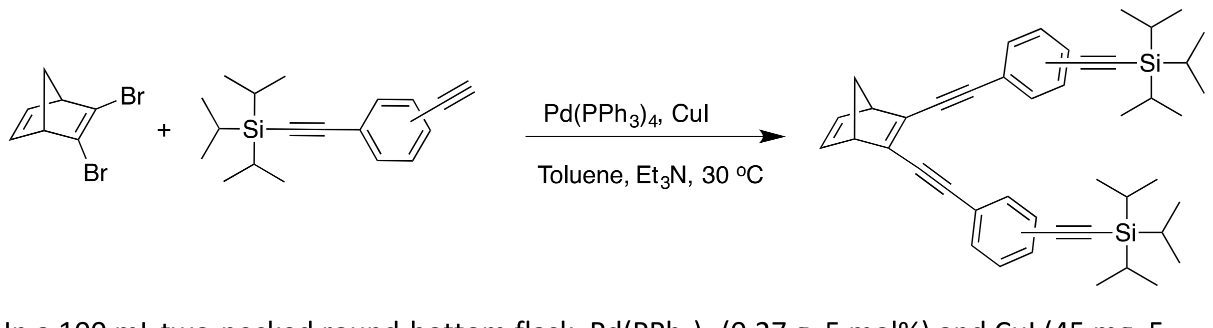

In a 100 mL two-necked round-bottom flask, $Pd(PPh_3)_4$ (0.27 g, 5 mol%) and CuI (45 mg, 5 mol %) were added. The flask was evacuated and refilled with nitrogen three times. Toluene (15 mL) was then added and the solution was stirred. 2,3-Dibromonorbornadiene* (0.59 g, 2.4 mmol) in 5 mL toluene was transferred to the reaction flask using a syringe, followed by the addition of **7** or **8** (4.8 mmol, 2 eq.) dissolved in toluene (5 mL). Triethylamine (3 mL),

purged with nitrogen beforehand, was added, and the reaction was stirred at 30 °C overnight. The solution was initially yellow which changed to reddish brown overnight. The solvents were removed using a rotary evaporator. The crude was washed with deionized water and extracted with dichloromethane, dried over anhydrous $MgSO_4$, filtered and the solvent was removed *in vacuo*. The reddish brown crude product was submitted to automated chromatography with a gradient of 0-10% dichloromethane in hexane, with a flow rate 50 mL/min to afford the desired products.

Meta-oligophenylethynyl-NBD (**9**): brown thick oil 700 mg (45%)

^1H NMR (400 MHz, $CDCl_3$) δ = 7.60 (td, *J* = 1.6, 1.6, 0.8 Hz, 2H), 7.41 (m, 4H), 7.26 (m, 4H), 6.87 (t, *J* = 1.9, 1.9 Hz, 2H), 3.78 (m, 2H), 2.30 (m, 2H), 2.19 (m, 2H), 1.13 (d, *J* = 0.5 Hz, 42H). The solvent $CDCl_3$ peak overlaps with part of the aromatic proton signals. ^{13}C NMR (101 MHz, $CDCl_3$) δ = 142.07, 141.87, 134.83, 131.91, 131.35, 128.44, 123.98, 123.79, 106.29, 102.62, 91.50, 86.51, 71.59, 56.32, 18.81, 11.45.

MS: MALDI-TOF: predicted 652.39 found: 651.89

Elemental analysis: Anal. Calcd for $C_{45}H_{56}Si_2$ C: 82.76, H: 8.64. Found C: 82.15, H: 8.55.

Para-oligophenylethynylNBD (**10**): fluffy orange solid 1.19 g (77%).

^1H NMR (400 MHz, $CDCl_3$) δ = 7.41 (m, 8H), 6.88 (t, *J* = 1.9, 1.9 Hz, 2H), 3.78 (m, 2H), 2.30 (dt, *J* = 6.7, 1.6, 1.6 Hz, 1H), 2.18 (dt, *J* = 6.7, 1.6, 1.6 Hz, 1H), 1.13 (d, *J* = 1.1 Hz, 42H). ^{13}C NMR (101 MHz, $CDCl_3$) δ = 142.12, 142.08, 132.13, 131.27, 123.53, 123.41, 106.87, 103.51, 93.11, 87.94, 71.55, 56.21, 18.82, 11.47.

MS: MALDI-TOF: predicted 652.39 found: 651.89

TBAF deprotection

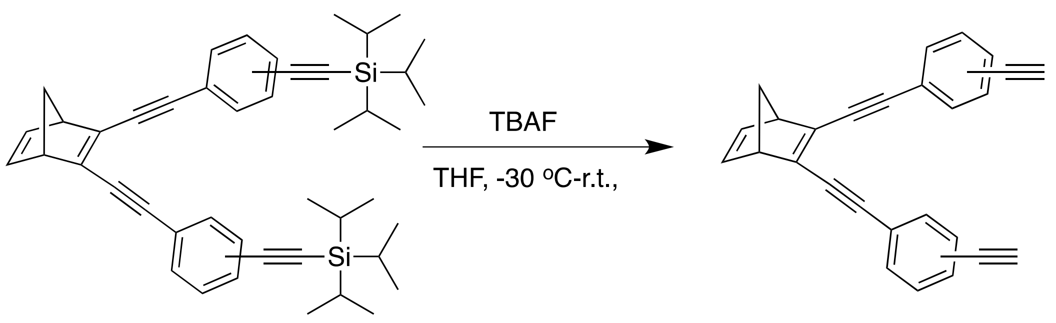

In 100 mL flask containing a magnetic stirbar, compound **9** or **10** (0.66 g, 1 mmol) was dissolved in THF (50 mL) and the solution was bubbled with nitrogen for 15 min. The flask was cooled to -30 °C with an acetonitrile/N_2 bath. Then, tributylammonium fluoride (1.0 M

solution in THF, 2.3 mL, 2.3 mmol) was added dropwise, while the yellow solution turned reddish. The reaction was stirred overnight at room temperature. The solvent was removed *in vacuo*, the crude product was washed with deionized water and brine, then extracted with dichloromethane and dried over anhydrous $MgSO_4$. After filtration, the solvent was removed *in vacuo*. The brown crude product was submitted to automated flash chromatography using a gradient of 0-20% dichloromethane in hexane (100 %). After removing the solvents *in vacuo*, the products **11** and **12** were collected.

11: brownish oil, 0.28 g (80%)

^{1}H NMR (400 MHz, $CDCl_3$) δ = 7.62 (td, *J* = 1.6, 1.6, 0.5 Hz, 2H), 7.44 (m, 4H), 7.29 (td, *J* = 7.8, 7.8, 0.6 Hz, 2H), 6.88 (m, 2H), 3.78 (dq, *J* = 2.3, 1.6, 1.6, 1.6 Hz, 2H), 3.08 (s, 2H), 2.31 (m, 1H), 2.18 (dt, *J* = 6.7, 1.6, 1.6 Hz, 1H).

^{13}C NMR (101 MHz, $CDCl_3$) δ = 142.08, 134.98, 132.00, 131.81, 128.59, 123.96, 122.62, 102.45, 86.65, 82.92, 77.92, 71.64, 56.30.

MS: MALDI-TOF: predicted 340.12 found: 339.90

12: light yellow oil which turns reddish upon storage, 0.30 g (86%).^{1}H NMR (400 MHz, $CDCl_3$) δ = 7.44 (m, 8H), 6.88 (t, *J* = 1.9, 1.9 Hz, 2H), 3.79 (m, 2H), 3.18 (s, 2H), 2.31 (dt, *J* = 6.8, 1.6, 1.6 Hz, 1H), 2.18 (dt, *J* = 6.8, 1.6, 1.6 Hz, 1H).^{13}C NMR (101 MHz, $CDCl_3$) δ = 142.14, 142.08, 132.21, 131.37, 124.01, 122.09, 103.15, 88.00, 83.45, 79.18, 71.59, 56.27.

MS: MALDI-TOF: predicted 340.12 found: 339.90

Final coupling of 3 or 4 *via* a Sonogashira reaction

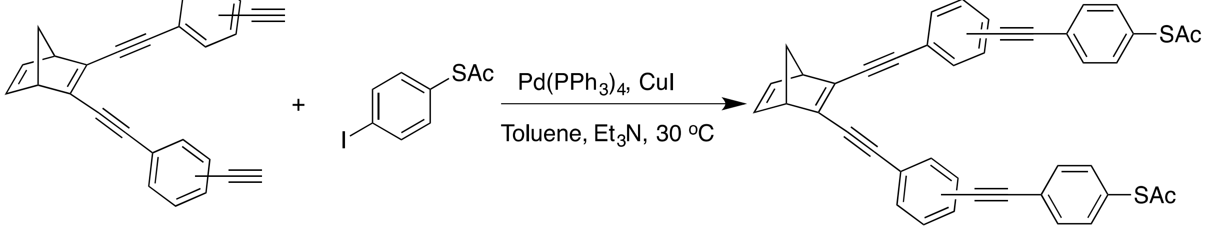

To a two-necked round-bottom flask equipped with a magnetic stirbar $Pd(PPh_3)_4$ (10 mol %), CuI (5 mol %) and 4-iodophenylthioacetate* (1.7 mmol) were added. The flask was evacuated and refilled with nitrogen three times. To the flask was added dry toluene (15 mL), followed by **11** or **12** dissolved in 10 mL toluene dropwise via a syringe. Then, degassed triethylamine (5 mL) was added to the red solution and the reaction mixture was stirred at 30 °C overnight. The solution was filtered through short silica gel plug using

dichloromethane as the eluent. The solution was concentrated and subjected to automated flash chromatography using a gradient of 0-30% dichloromethane in hexane. The product was collected after removing the solvent *in vacuo*. Recrystallization from acetonitrile yield analytically pure product.

3: light brown fluffy solid 91 mg (17%).

^{1}H NMR (400 MHz, $CDCl_3$) δ 7.67 (td, *J* = 1.7, 1.7, 0.6 Hz, 2H), 7.53 (m, 4H), 7.47 (m, 4H), 7.38 (m, 4H), 7.33 (ddd, *J* = 8.0, 7.4, 0.6 Hz, 2H), 6.89 (m, 2H), 3.79 (dt, *J* = 3.3, 1.6, 1.6 Hz, 2H), 2.44 (s, 6H), 2.33 (dt, *J* = 6.7, 1.7, 1.7 Hz, 1H), 2.19 (dt, *J* = 6.8, 1.6, 1.6 Hz, 1H). ^{13}C NMR (101 MHz, $CDCl_3$) δ 193.52, 142.09, 141.98, 134.52, 134.36, 132.35, 131.56, 131.53, 128.67, 128.41, 124.04, 123.43, 102.59, 90.33, 89.35, 86.72, 71.64, 56.33, 30.44.

ATR-IR: 3059.7, 2938.4, 1706.7.

Elemental analysis: Anal. Calcd for $C_{43}H_{28}O_2S_2$ C: 80.60, H: 4.40, S: 10.01. Found C: 80.13, H: 4:61, S: 9.59.

HRMS: calculated 640.1531; [M+H$^+$] Found 641.1600

4: bright yellow solid (220 mg, yield 42 %).

^{1}H NMR (400 MHz, $CDCl_3$) δ = 7.55 (m, 4H), 7.49 (m, 8H), 7.40 (m, 4H), 6.89 (m, 2H), 3.80 (dt, *J* = 3.1, 1.6, 1.6 Hz, 2H), 2.44 (s, 6H), 2.32 (dt, *J* = 6.8, 1.7, 1.7 Hz, 1H), 2.19 (dt, *J* = 6.8, 1.6, 1.6 Hz, 1H). ^{13}C NMR (101 MHz, $CDCl_3$) δ = 193.52, 142.13, 142.09, 134.38, 132.32, 131.78, 131.48, 128.44, 124.43, 123.70, 122.92, 110.16, 103.46, 90.97, 90.77, 88.17, 71.56, 56.30, 30.45.

ATR-FTIR: 2950, 1699, 1502, 834.

Elemental analysis: Anal. Calcd for $C_{43}H_{28}O_2S_2$ C: 80.60, H: 4.40. Found C: 79.86, H: 4:00.

MS: MALDI-TOF: predicted 640.15 found: 639.95

Thiophene coupling to NBD *via* Sonogashira reaction

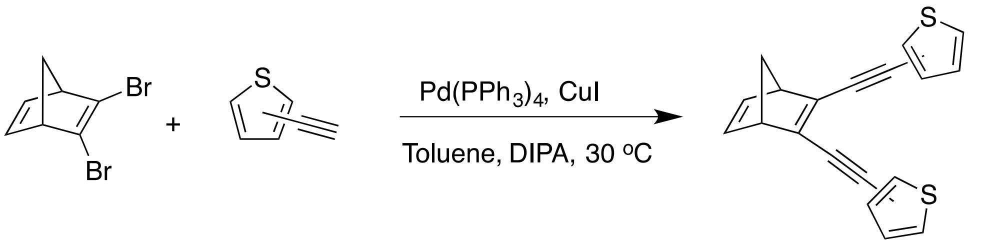

In an oven-dried Schlenk flask (25 mL) provided with magnetic stirring bar, were added $Pd(PPh_3)_4$ (5 mol%), CuI (10 mol%), 2,3-dibromonorbornadiene* (1.2 mmol) and toluene (10

mL). The solution was purged with nitrogen for 15 minutes. Then, diisopropylamine (1 mL) and 2- or 3-ethynylthiophene (2.6 mmol) were added, at which point the color of the solution turned from yellow to dark red. The mixture was reacted until the starting materials were consumed as indicated by TLC. The solution was then diluted with dichloromethane and filtered through a short silica gel plug. The volatiles were then removed using a rotary evaporator. The crude product obtained was submitted to automated flash chromatography using an eluent gradient of 0-5% dichloromethane in hexane. The product was obtained after the solvent was removed *in vacuo*.

2: Off-white solid 220 mg (60 %)

^{1}H NMR (400 MHz, $CDCl_3$) δ = 7.48 (dt, *J* = 3.0, 0.9, 0.9 Hz, 2H), 7.28 (ddd, *J* = 5.0, 3.0, 0.6 Hz, 2H), 7.16 (dt, *J* = 5.0, 0.9, 0.9 Hz, 2H), 6.87 (t, *J* = 2.0, 2.0 Hz, 2H), 3.76 (h, *J* = 1.6, 1.6, 1.6, 1.6, 1.6 Hz, 2H), 2.29 (dt, *J* = 6.7, 1.7, 1.7 Hz, 1H), 2.16 (dt, *J* = 6.7, 1.6, 1.6 Hz, 1H). ^{13}C NMR (101 MHz, $CDCl_3$) δ = 142.04 , 141.21 , 129.88 , 128.53 , 125.47 , 122.71 , 98.20 , 85.56 , 71.45 , 56.23 .

ATR-IR: 3106.4, 2986.1, 2180.8, 778.4.

Elemental analysis: Anal. Calcd for $C_{43}H_{28}O_2S_2$ C: 74.96, H: 3.97. Found C: 75.34, H: 3.87.

HRMS: calculated 304.0380; [M+H$^+$] Found 305.0451

1: yellow solid 0.20 g (55 %)

^{1}H NMR (400 MHz, $CDCl_3$) δ = 7.31 (m, 1H), 7.25 (dd, *J* = 3.7, 1.2 Hz, 1H), 7.01 (m, 1H), 6.87 (m, 1H), 3.78 (dq, *J* = 2.2, 1.6, 1.6, 1.6 Hz, 1H), 2.29 (ddd, *J* = 6.7, 1.9, 1.4 Hz, 1H), 2.16 (dt, *J* = 6.7, 1.6, 1.6 Hz, 1H). ^{13}C NMR (101 MHz, $CDCl_3$) δ = 142.03, 141.18, 131.94, 127.93, 127.39, 123.62, 96.77, 90.01, 71.31, 56.11.

ATR-IR: 3093.8(w), 2986.7(w), 2173.0(w), 698.0(s)

Elemental analysis: Anal. Calcd for $C_{43}H_{28}O_2S_2$ C: 74.96, H: 3.97. Found C: 74.7, H: 4.14.

HRMS: calculated 304.0380; [M+H$^+$] Found 305.0451

*Synthesized according to literature procedure.[2]

II. Nuclear Magnetic Resonance (NMR) data

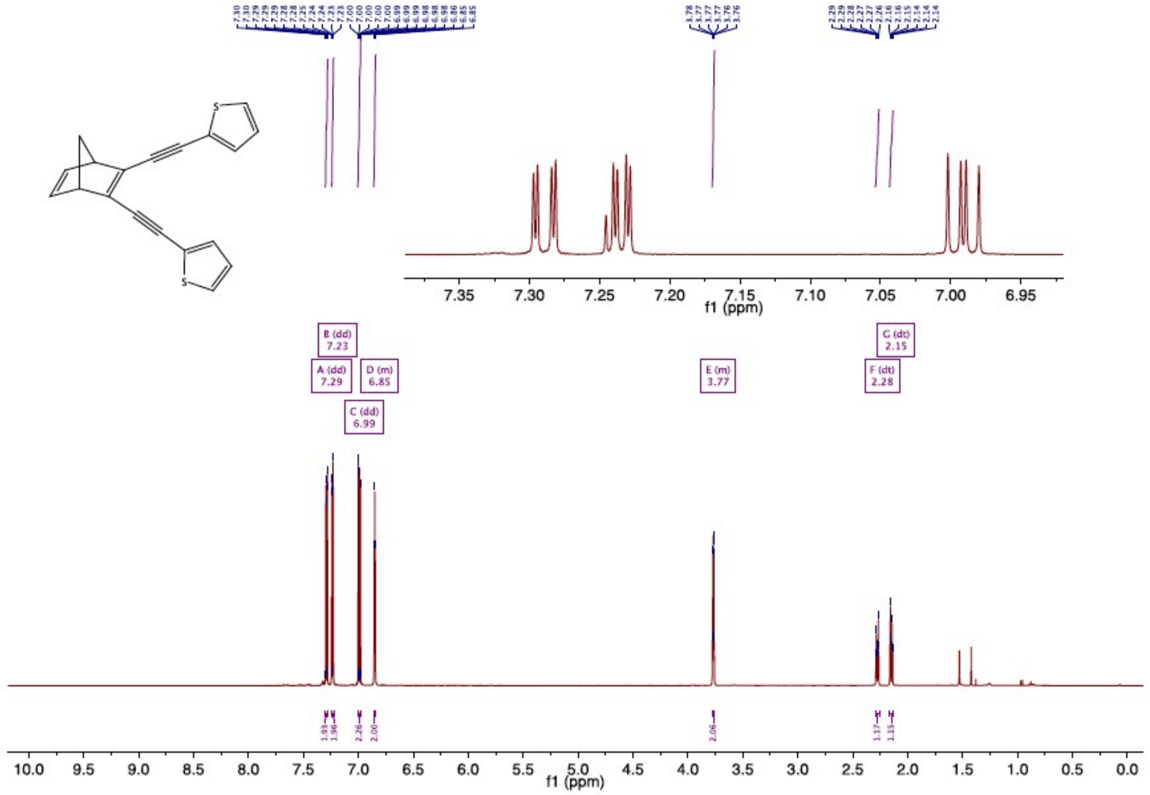

*Figure S1: ^{1}H-NMR (400 MHz) of **1** in $CDCl_3$.*

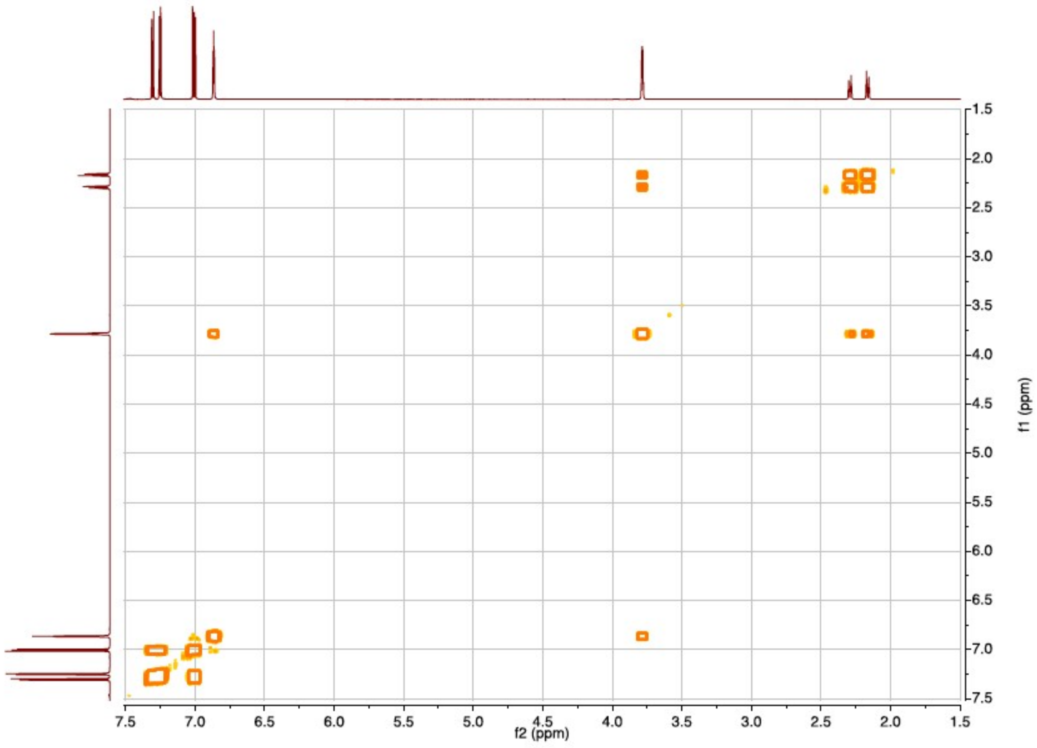

*Figure S2: COSY-NMR (400 MHz) of **1** in $CDCl_3$.*

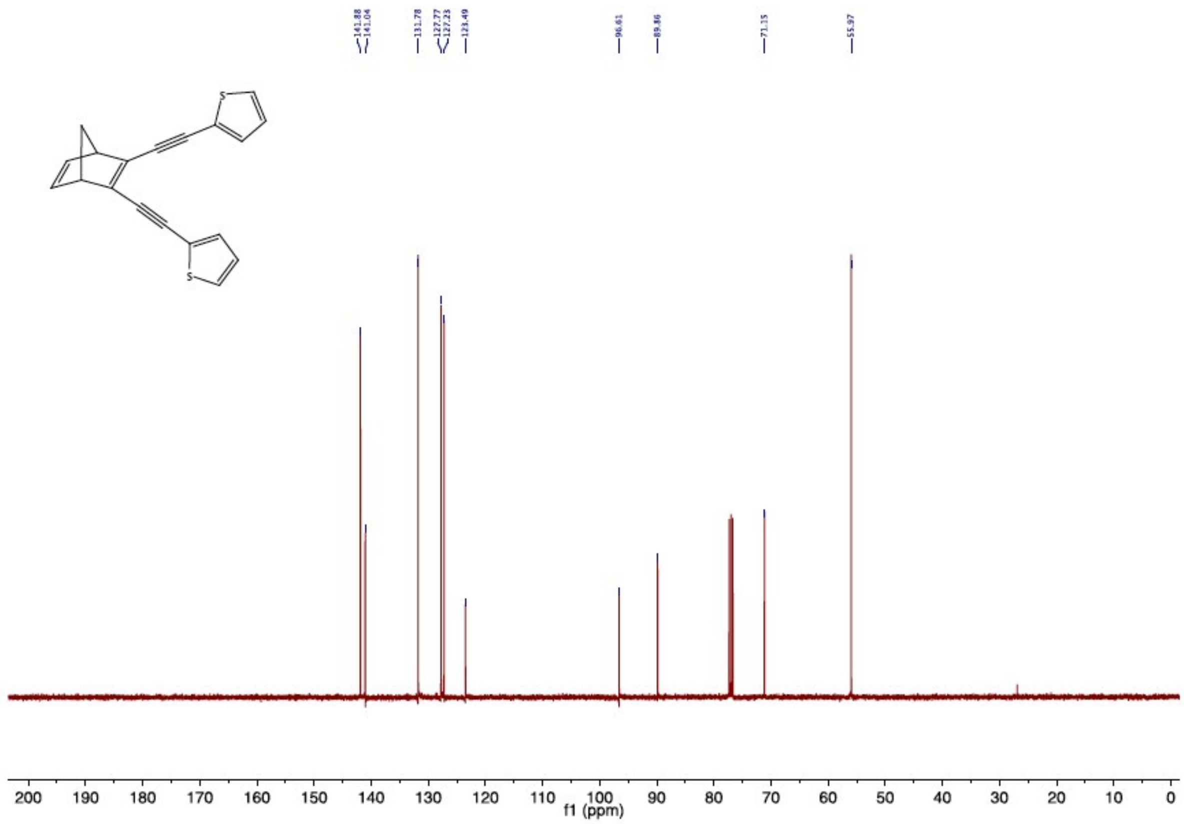

*Figure S3: ^{13}C-NMR (101 MHz) of **1** in $CDCl_3$.*

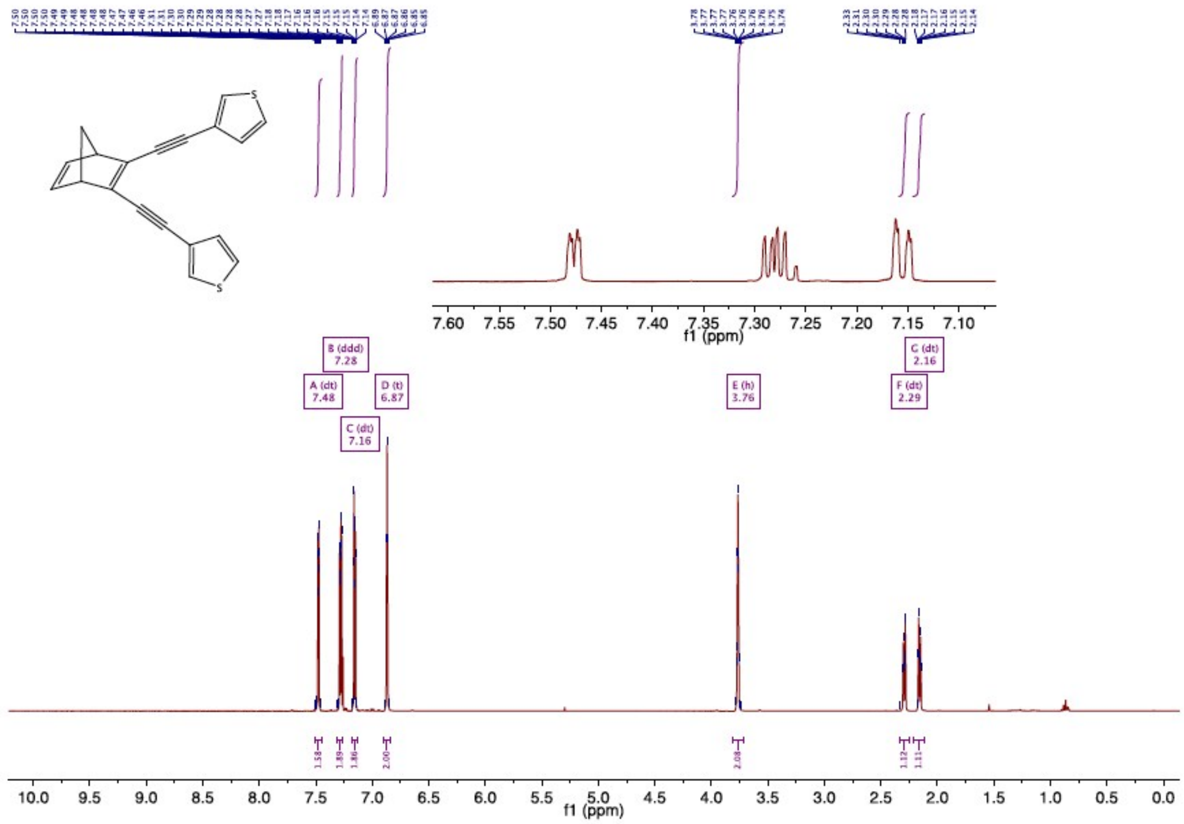

*Figure S4: ^{1}H-NMR (400 MHz) of **2** in $CDCl_3$.*

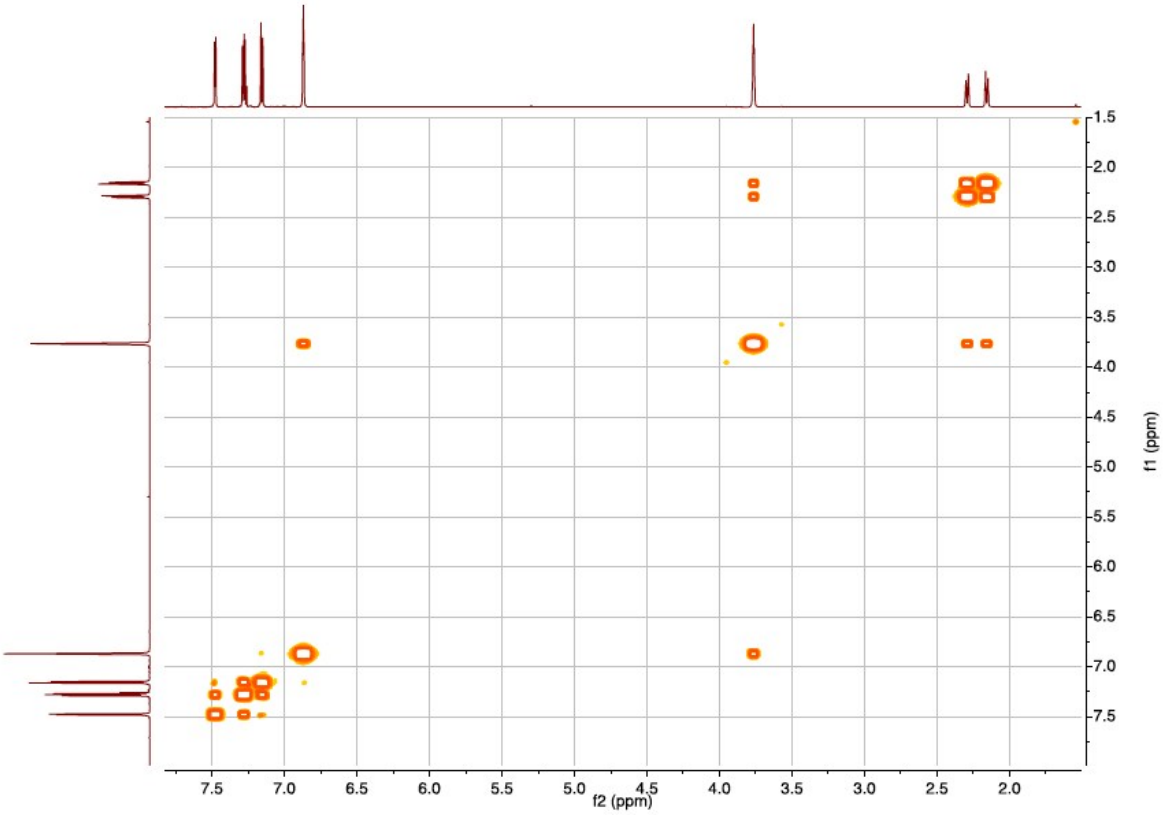

*Figure S5: COSY-NMR (400 MHz) of **2** in $CDCl_3$.*

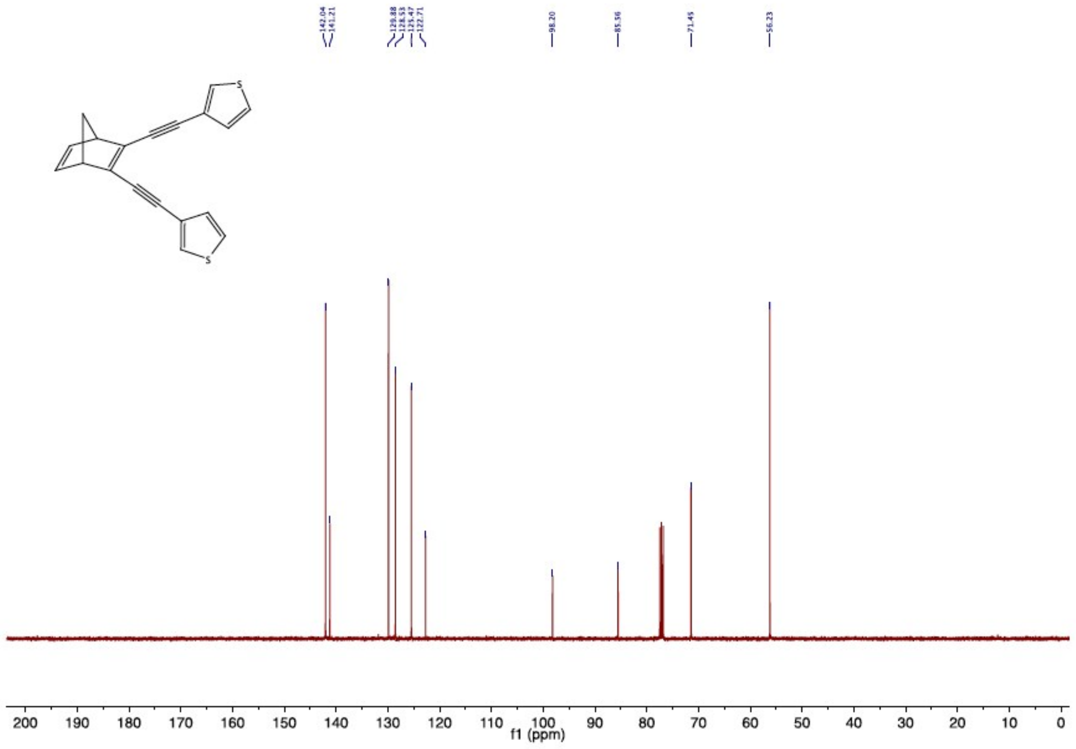

*Figure S6: ^{13}C-NMR (101 MHz) of **2** in $CDCl_3$.*

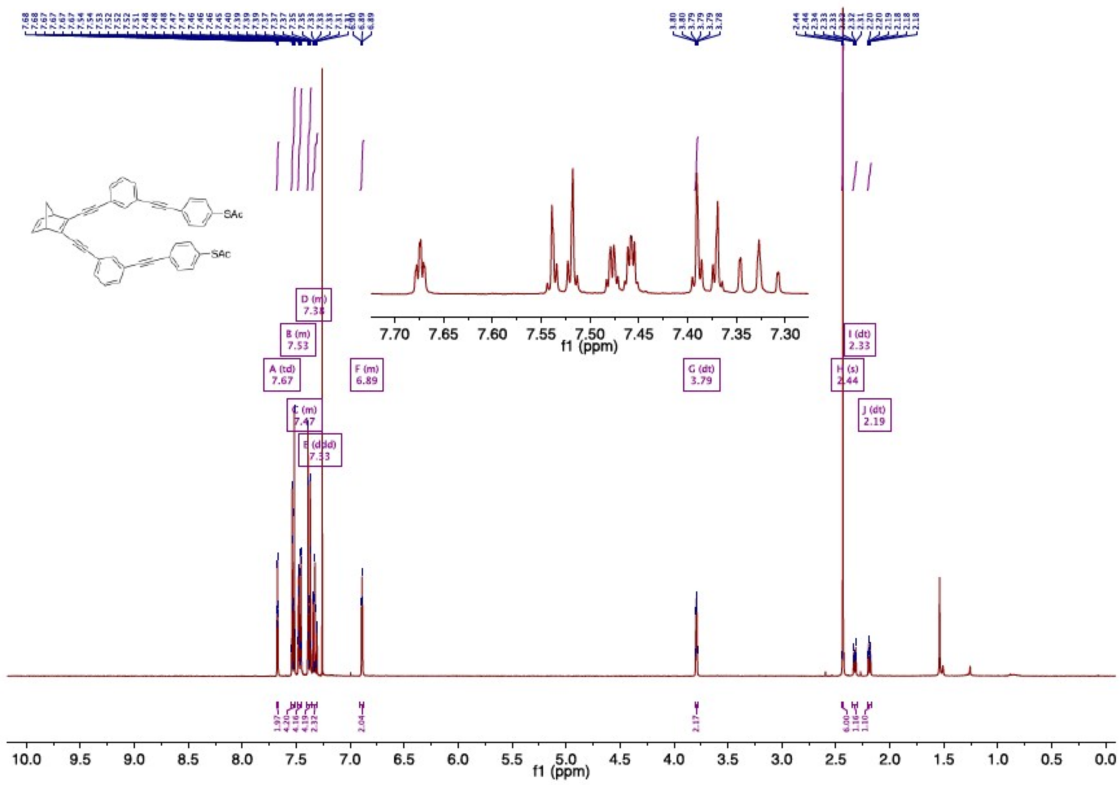

*Figure S7: ^{1}H-NMR (400 MHz) of **3** in $CDCl_3$.*

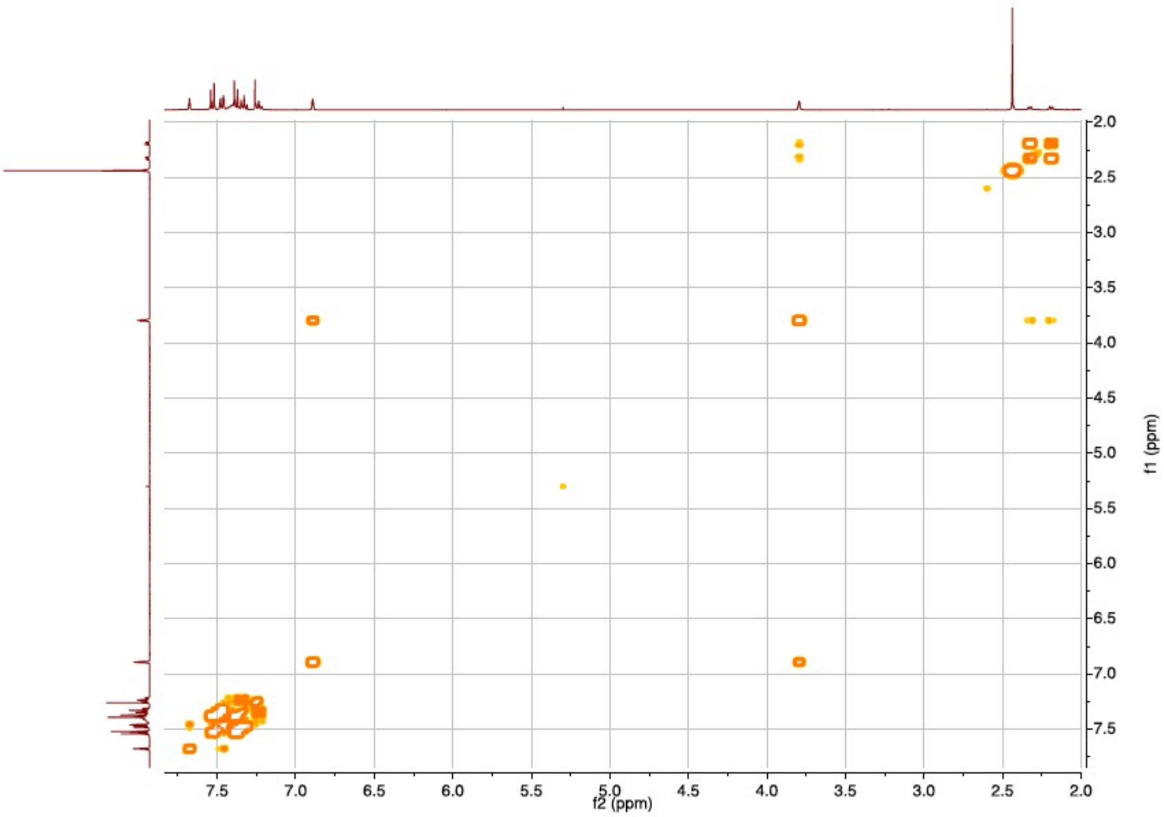

*Figure S8: COSY-NMR (400 MHz) of **3** in $CDCl_3$.*

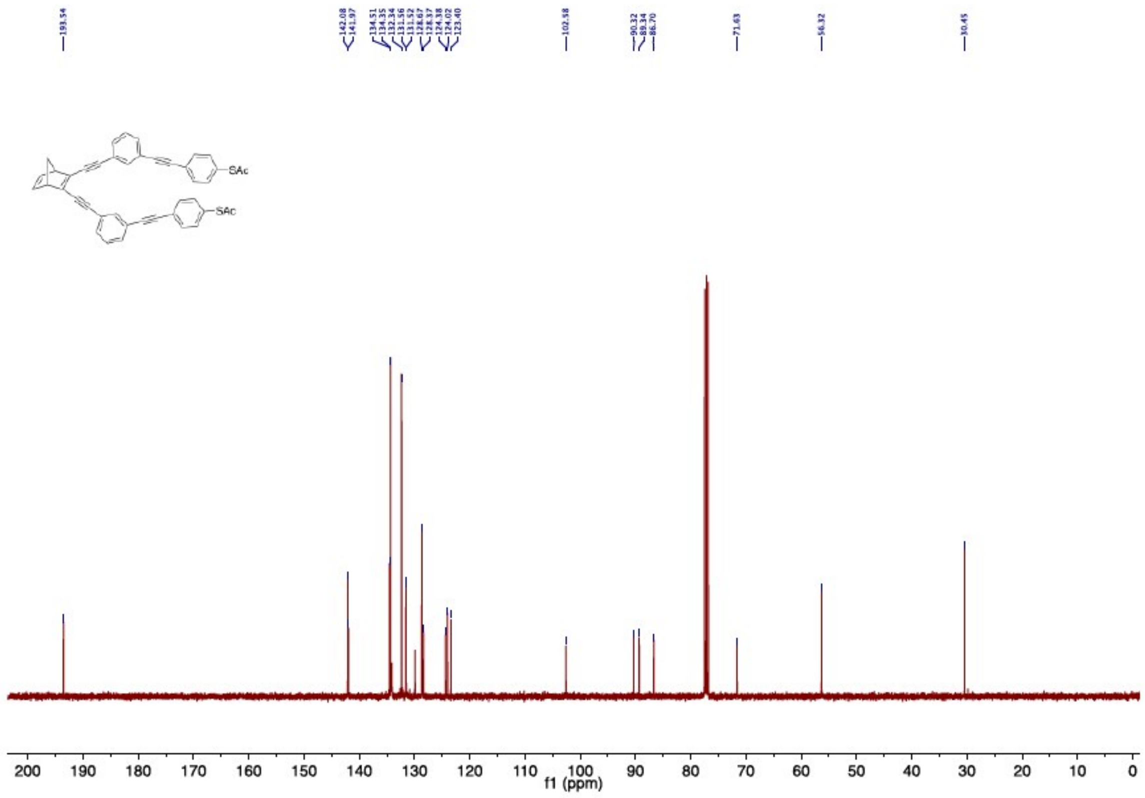

*Figure S9: ^{13}C-NMR (101 MHz) of **3** in $CDCl_3$.*

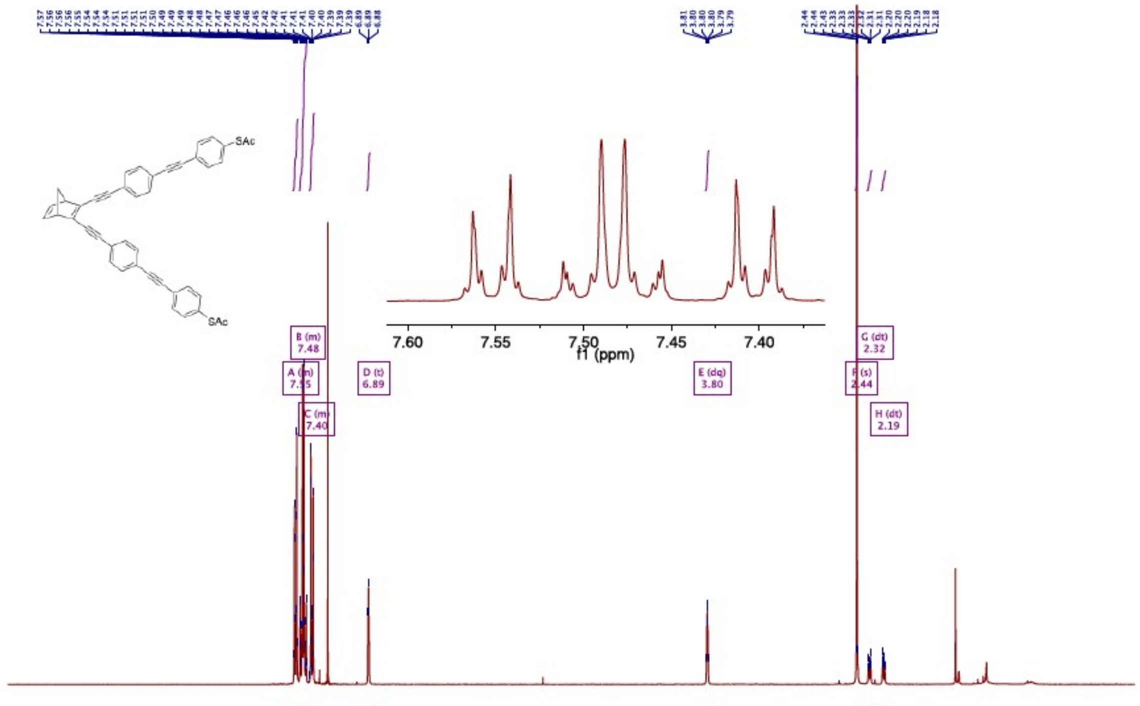

*Figure S10: ^{1}H-NMR (400 MHz) of **4** in $CDCl_3$.*

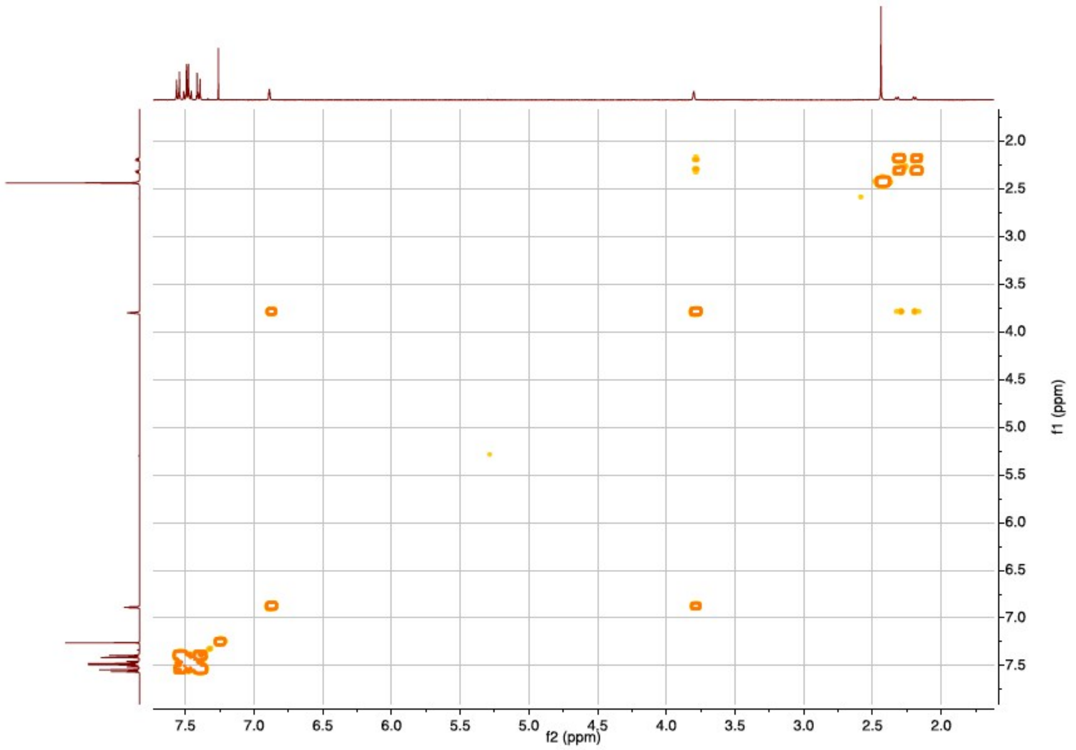

*Figure S11: COSY-NMR (400 MHz) of **4** in $CDCl_3$.*

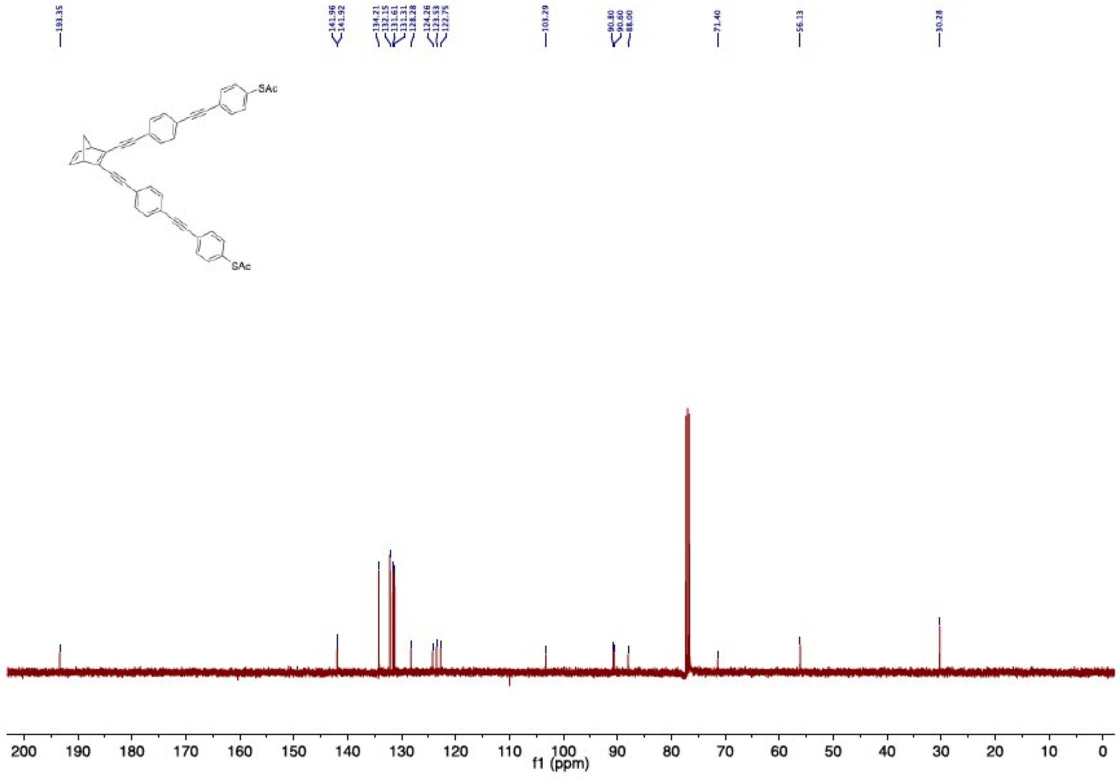

*Figure S12: ^{13}C-NMR (101 MHz) of **4** in $CDCl_3$.*

III. Ultraviolet-visible (UV-vis) spectrophotometric study

Molar absorptivity

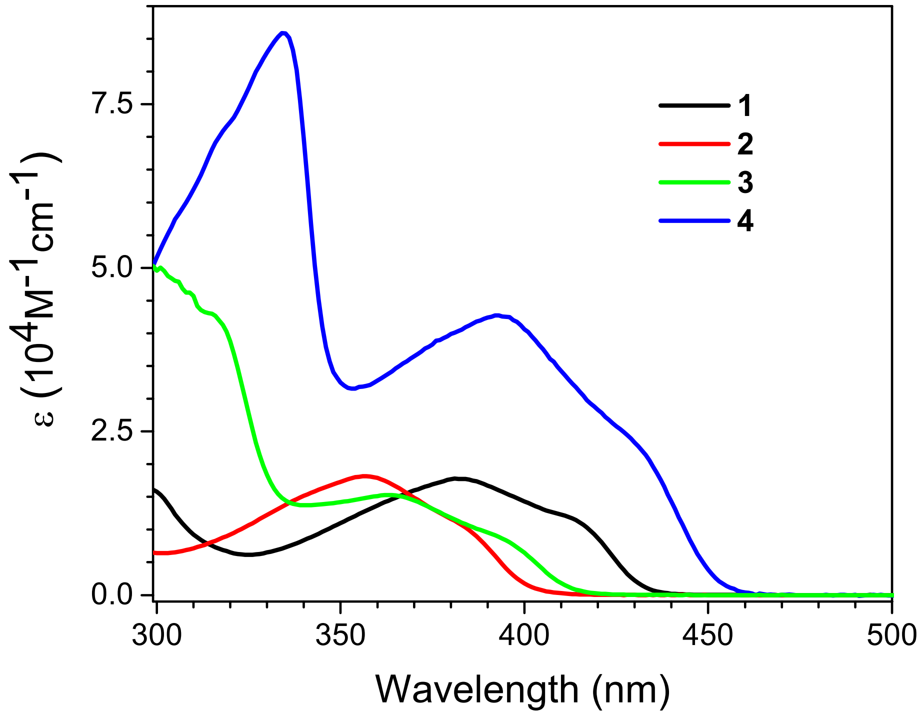

*Figure S13: Molar absorptivity vs wavelength of compounds **1**, **2**, **3** and **4**. Three samples dissolved in toluene were taken for each measurement. The average value is displayed.*

Kinetics

Arrhenius Plot

Using the Arrhenius equation $\ln k = \ln A - \frac{E_a}{RT}$ and plotting ln*k* vs 1/T gave a linear correlation. The slope correspond to the activation energy E_a while the intercept corresponds to the pre-exponential factor, A.

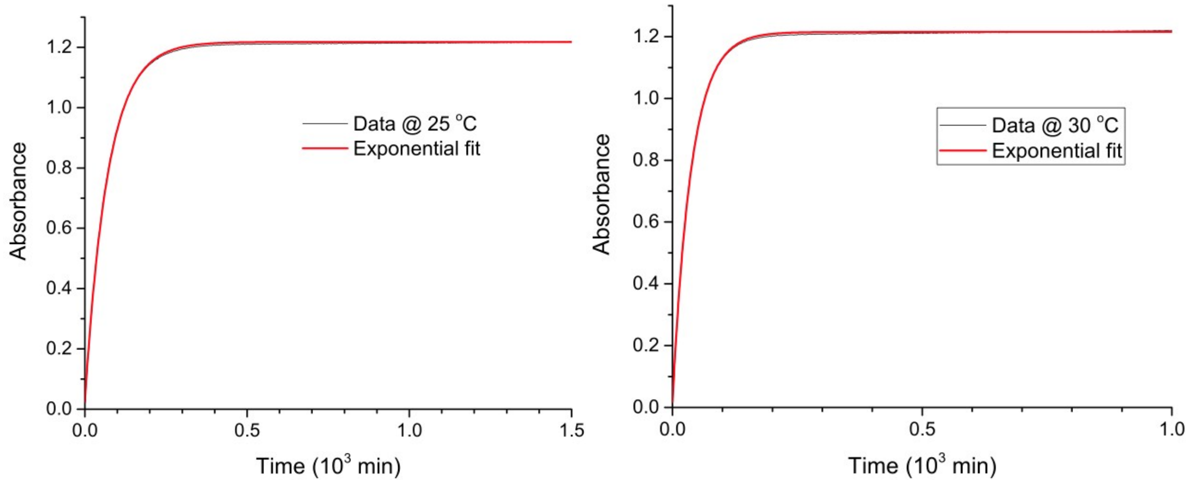

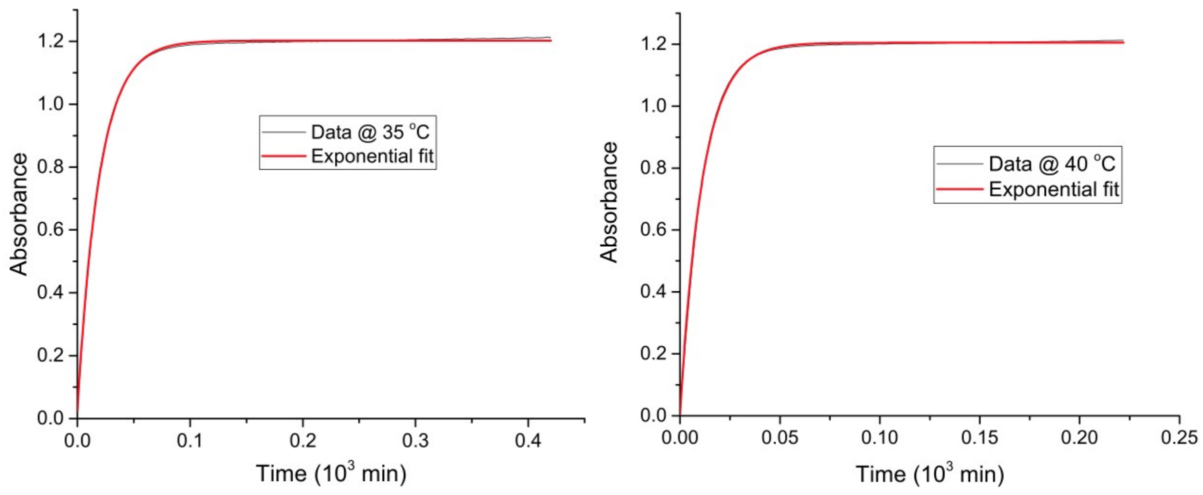

*Figure S14: Thermal recovery of the absorption (λ_{max} = 381 nm) of **1** at 25 °C (a), 30 °C (b), 35 °C (c) and 40 °C (d). The data is fit exponentially to extract the rate constant, k, at the corresponding temperatures.*

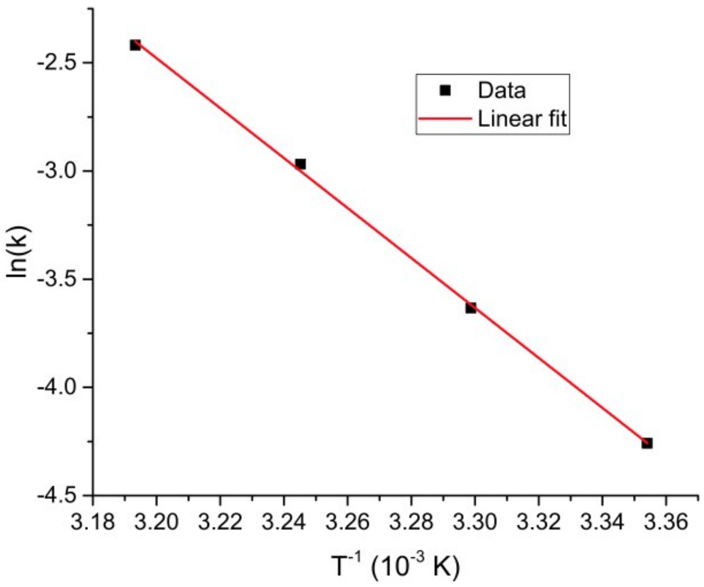

*Figure S15: Arrhenius plot for **1** gave the following values for the Arrhenius parameters. A = 4.0 $\times$ 10^{14} and E_a = 93.8 kJ/mol, respectively.*

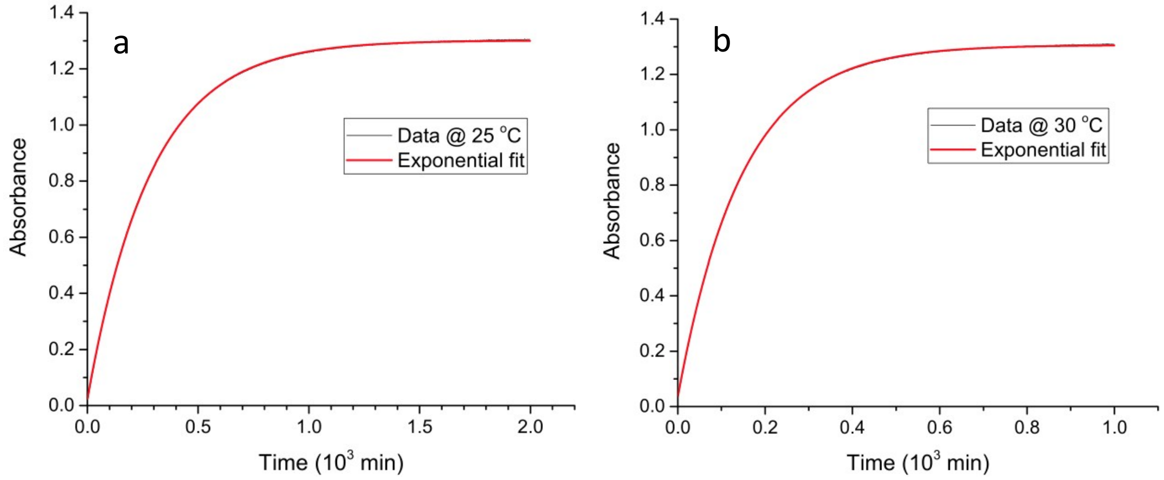

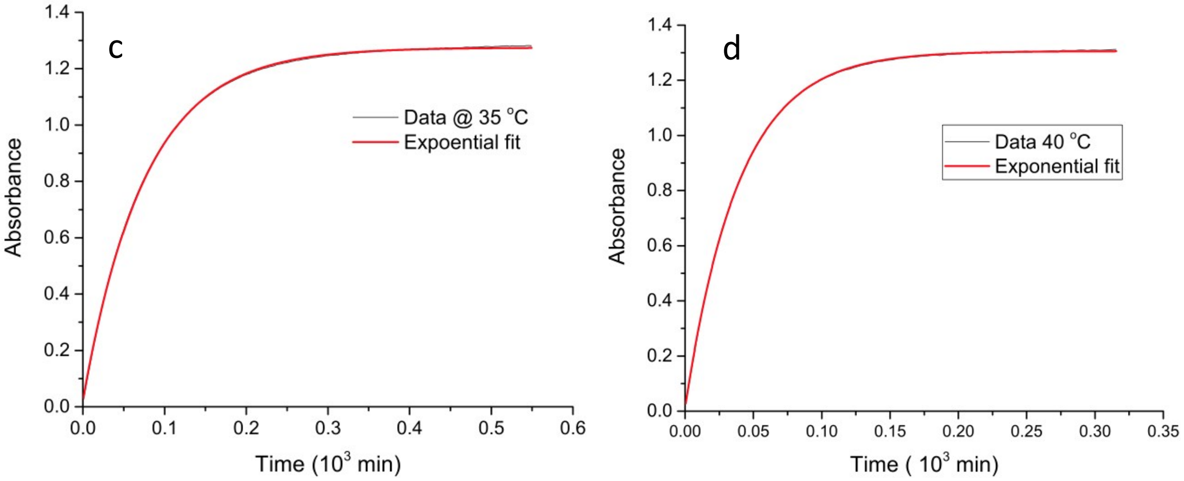

*Figure S16: Thermal recovery of the absorption ($\square_{max}$ = 357 nm) of **2** at 25 °C (a), 30 °C (b), 35 °C (c) and 40 °C (d). The data is fit exponentially to extract the rate constant, k, at the corresponding temperatures.*

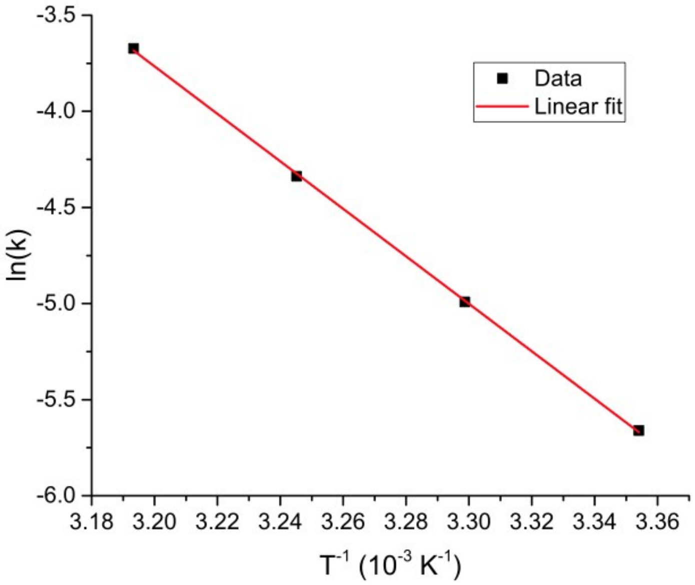

*Figure S17: Arrhenius plot for **2** gave the following values for the Arrhenius parameters. A = 5.0 $\times 10^{15}$ and E_a = 103.7 kJ/mol, respectively.*

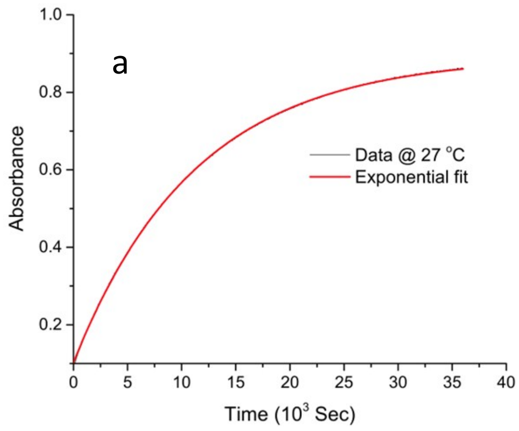

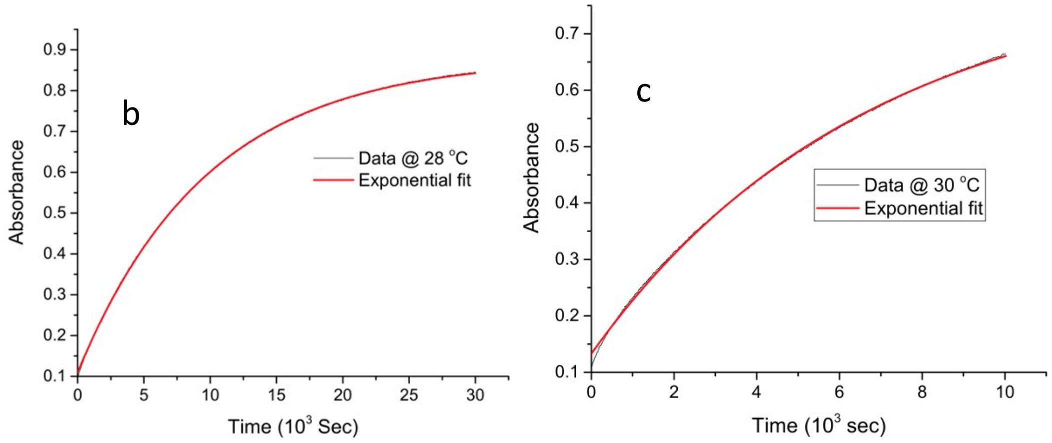

*Figure S18: Thermal recovery of the absorption (▯$_{max}$ = 363 nm) of **3** at 27 °C (a), 28 °C (b) and 30 °C (c). The data are fit exponentially to extract the rate constant, k, at the corresponding temperures.*

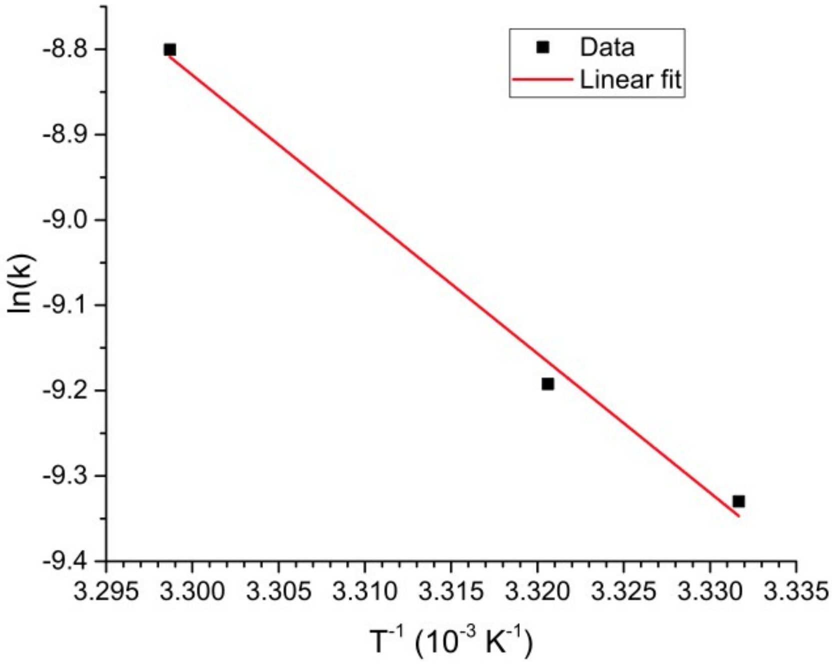

*Figure S19: Arrhenius plot for **3** gave the following values for the Arrhenius parameters. A = 3.5 $\times 10^{19}$ and E_a = 135.6 kJ/mol, respectively.*

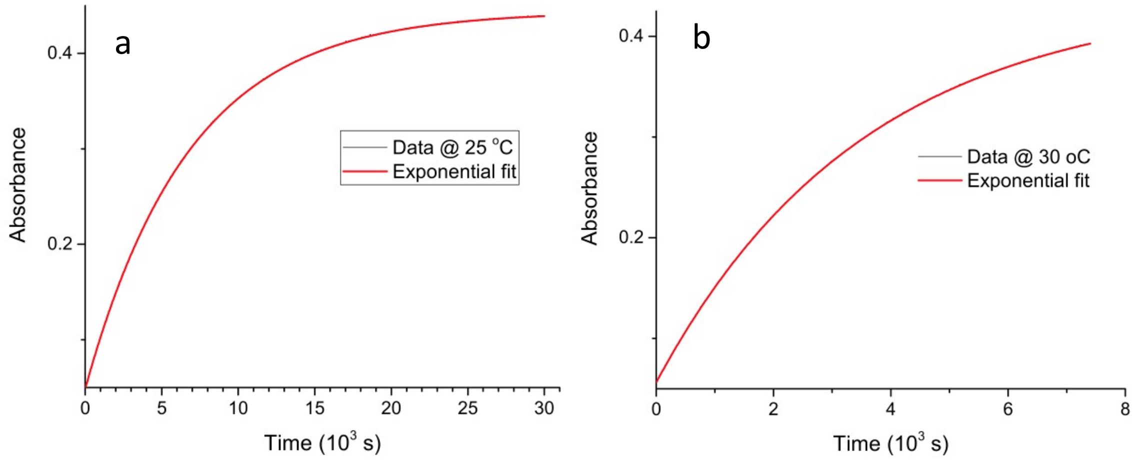

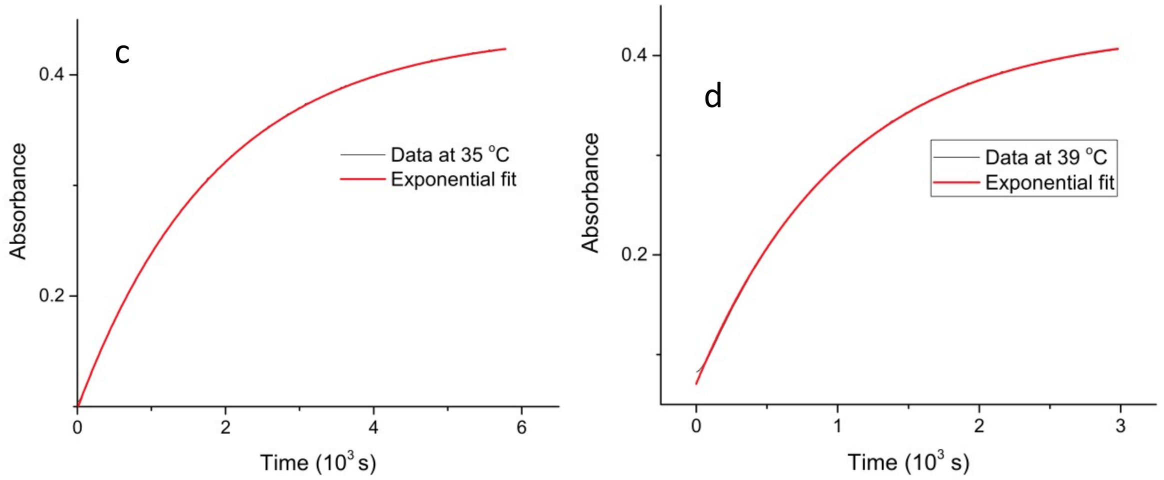

*Figure S20: Thermal recovery of the absorption (λ_{max} = 392 nm) of **4** at 25 °C (a), 30 °C (b), 35 °C (c) and 39 °C (d). The data is fit exponentially to extract the rate constant, k, at the corresponding temperatures.*

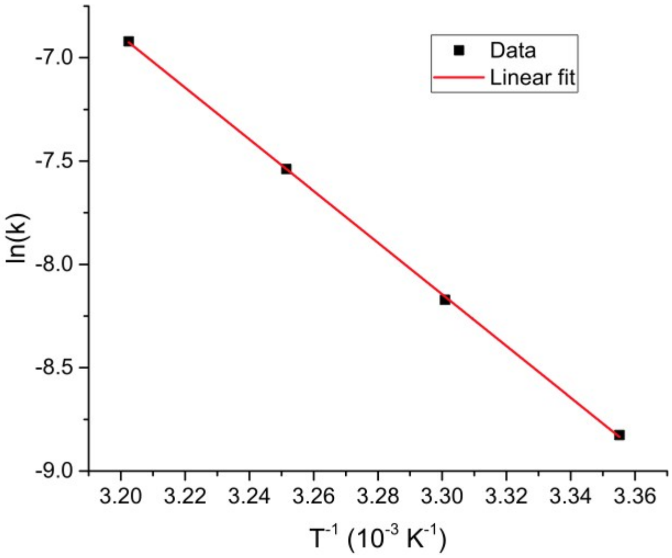

*Figure S21: Arrhenius plot for **4** gave the following values for the Arrhenius parameters. A = 1.6×10^{14} and E_a = 100.7 kJ/mol, respectively.*

IV. Photoisomerization quantum yield determination using ferrioxalate actinometry

Photon flux

The photon flux of the 365 nm and 405 nm LED lamps were measured using the ferrioxalate actinometry based on literature proedure.[3] To five volumetric flasks containing a phenanthroline solution in an acid buffer were added ferrioxalate solutions, which are irradiated for different times (0, 15, 30, 45, 60 s). The absorbance of tris-phenanthroline iron (II) complex at 510 nm as a function of irradiation time of the five solutions (black squares) was plotted. The points were fitted linearly to provide a slope, which is used to calculate the photon flux based on equation described in literature.[4]

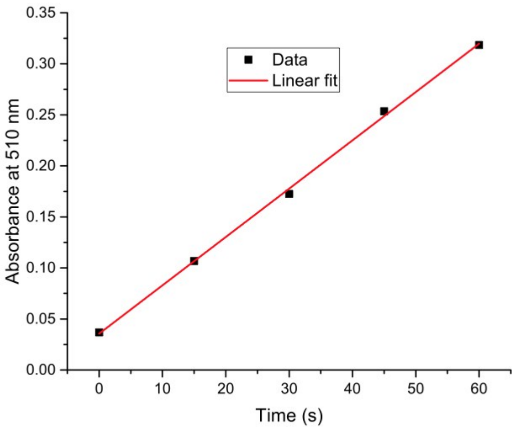

*Figure S22: The photon flux (11.71 $\times 10^{-9}$ E/s) of 405 nm lamp used for the photoisomerization of **1**.*

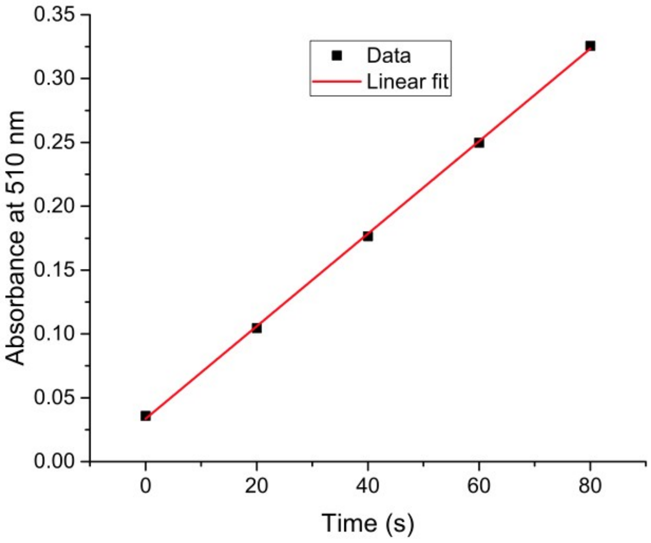

*Figure S23: The photon flux (7.93 $\times 10^{-9}$ E/s) of 365 nm lamp used for the photoisomerization of **2** and **3**.*

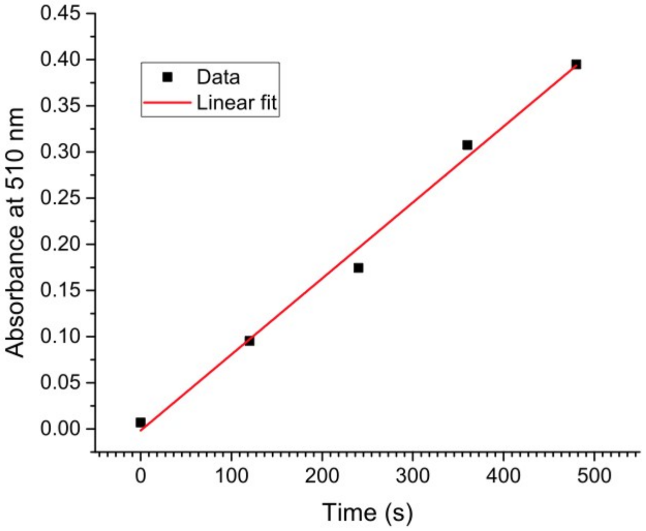

*Figure S24: The photon flux (9.01 $\times 10^{-9}$ E/s) of 405 nm lamp used for the photoisomerization of **4**.*

Photoisomerization quantum yield

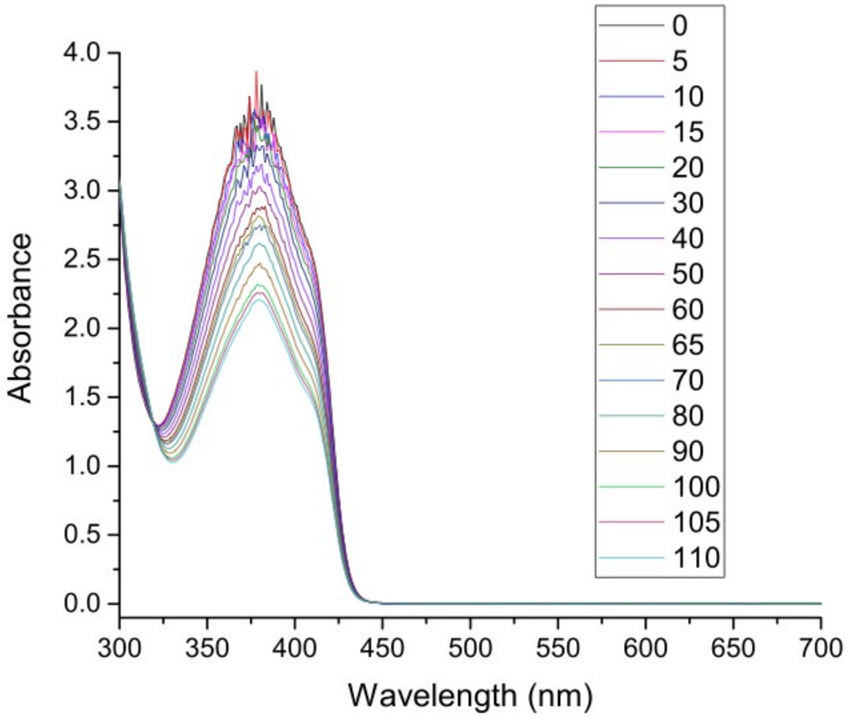

*Figure S25: The photoisomerization of **1** with a 365 nm lamp (photon flux Figure S23) to determine the quantum yield.*

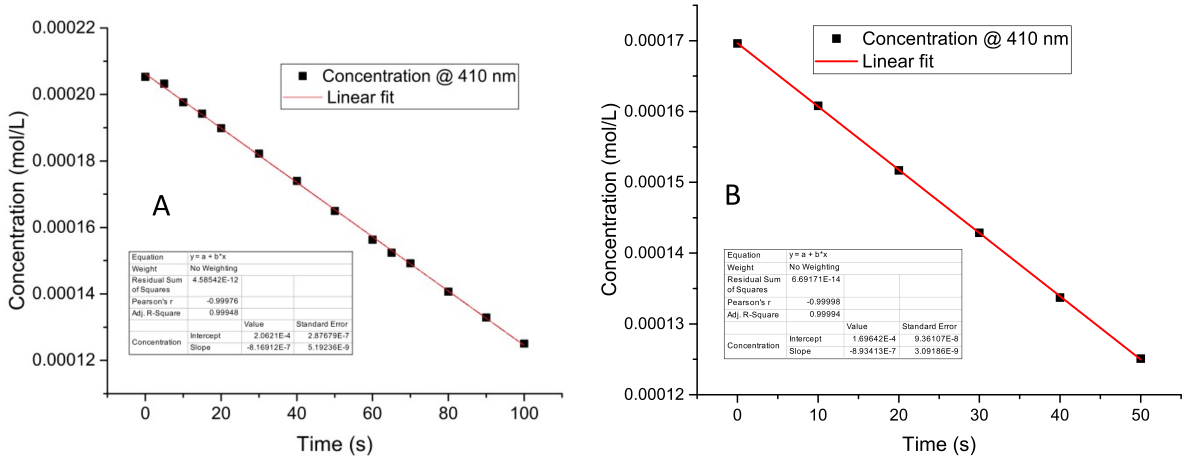

*Figure S26: The change in concentration of **1** at 410 nm at different irradiation times was plotted. The slope was used to calculate the quantum yield using the following equation* $\Phi = \frac{Slope\ * Sample\ volume}{Photon\ flux}$*. The quantum yield value is reported as the average of two (A and B) measurements (0.269 + 0.322)/2 = 0.296 ±0.035.*

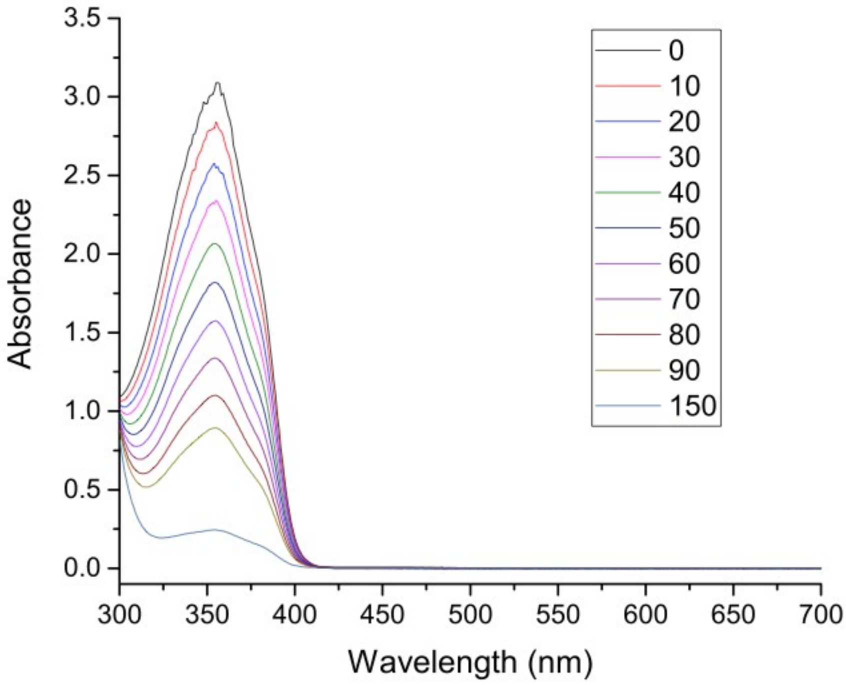

*Figure S27: The photoisomerization of **2** with a 365 nm lamp (photon flux Figure S24) to determine the quantum yield.*

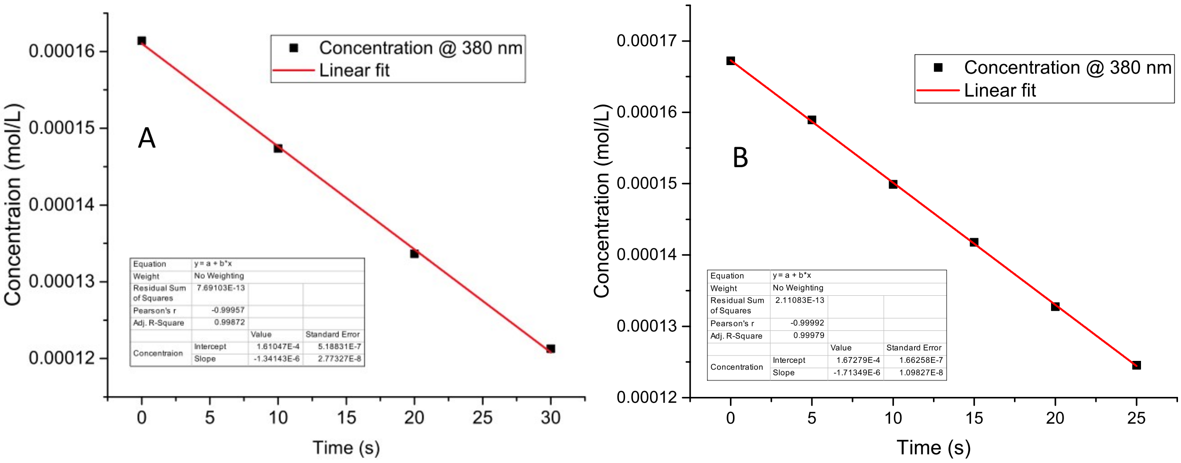

*Figure S28: The change in concentration of **2** at 380 nm at different irradiation times was plotted. The slope was used to calculate the quantum yield using the following equation* $\Phi = \frac{Slope\ * Sample\ volume}{Photon\ flux}$. *The quantum yield value is reported as the average of two (A and B) measurements (0.535 + 0.554)/2 = 0.544 ±0.007.*

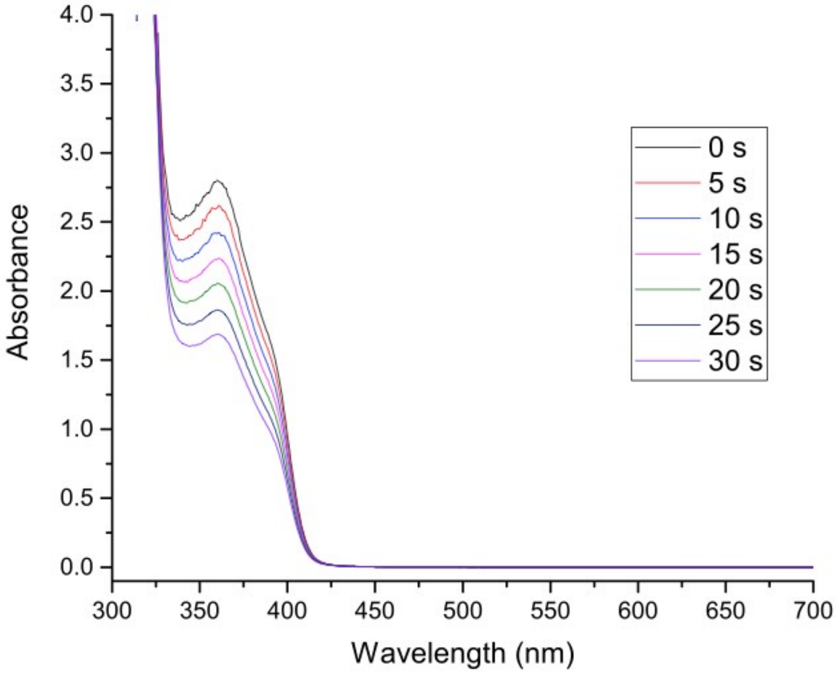

*Figure S29: The photoisomerization of **3** with a 365 nm lamp (photon flux Figure S24) to determine the quantum yield.*

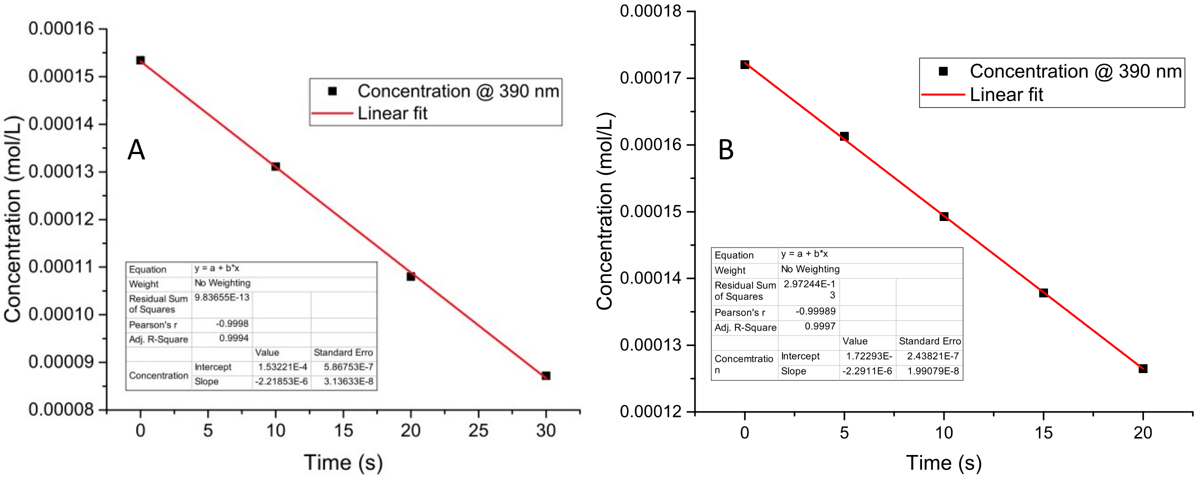

*Figure S30: The change in concentration of **3** at 390 nm at different irradiation times was plotted. The slope was used to calculate the quantum yield using the following equation* $\Phi = \frac{Slope \; * Sample\ volume}{Photon\ flux}$. *The quantum yield value is reported as the average of two (A and B) measurements (0.792 + 0.745)/2 = 0.768 ±0.028.*

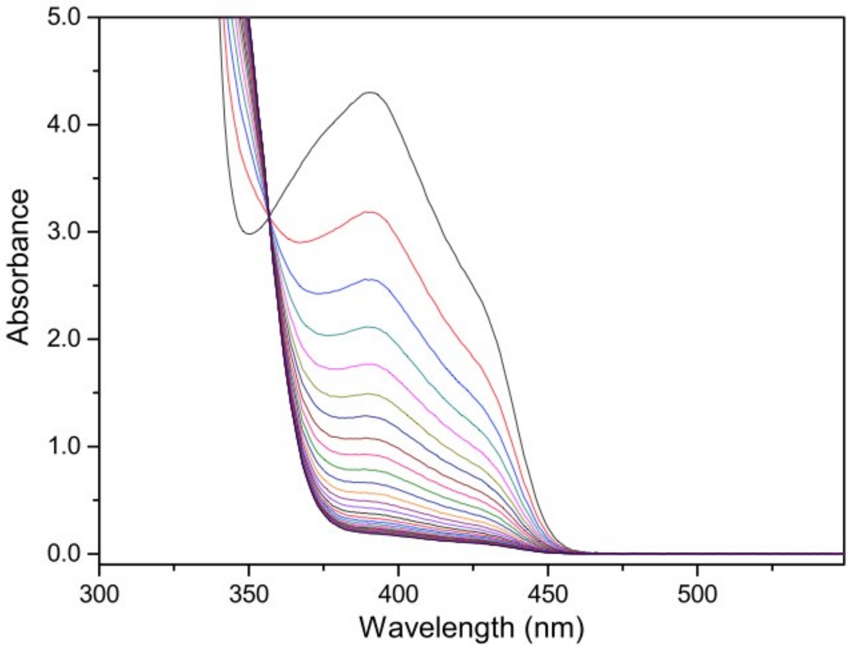

*Figure S31: The photoisomerization of **4** with a 365 nm lamp (photon flux Figure S22) to determine the quantum yield.*

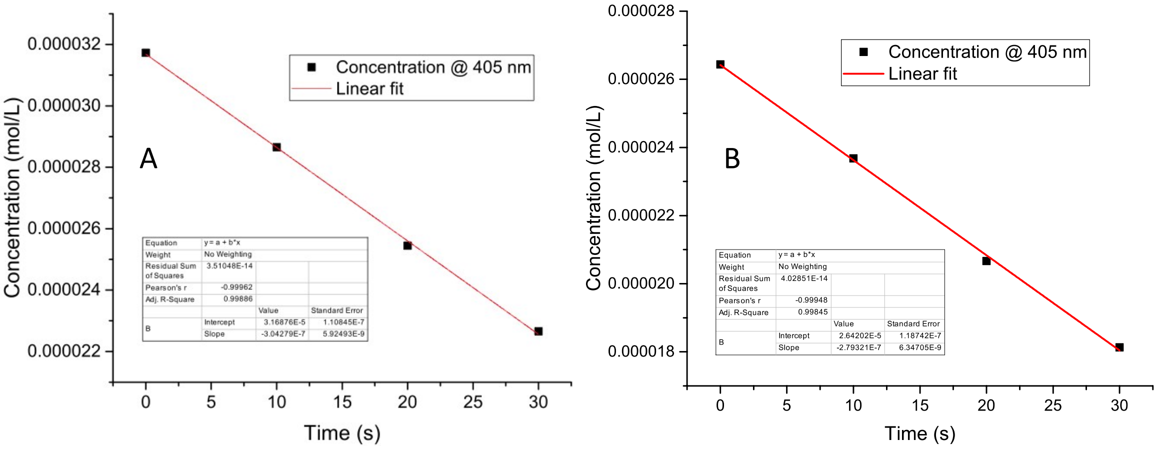

*Figure S32: The change in concentration of **4** at 405 nm at different irradiation times was plotted. The slope was used to calculate the quantum yield using the following equation* $\Phi = \frac{Slope\ * Sample\ volume}{Photon\ flux}$. *The quantum yield value is reported as the average of two measurements (0.150 + 0.149)/2 = 0.15 ±0.001.*

V. UV-vis absorption and emission measurements

Emission decay

*Table 1: Summary of fluorescence quantum yield,[5] measured time constants, relative amplitudes and χ^2 of curve fit. For **3**, the emission spectrum was corrected for reabsorption for by normalizing it to a spectrum of a dilute sample before QY calculation. Raman peaks in the emission spectrum for the compounds with low fluorescence QY were removed before QY calculation.*

**Compound**	Φ_F	τ_1 (ns)	A_1	τ_2	A_2	χ^2
**1**	0.0394 ± 0.16	0.21 ± 0.00089	1	-	-	1.33
**2**	0.0016 ± 0.01	0.020 ± 0.00033	0.9963	1.11	0.0037	1.78
**3**	0.0089 ± 0.11	0.023 ± 0.000524	0.9627	1.11	0.0373	1.57
**4**	0.4885 ± 1.45	1.28 ± 0.00515	1	-	-	1.39

TDDFT calculations

TDDFT//B3LYP/6-31G** excited state calculations on DFT//B3LYP/6-31G** optimized structures. Results for the ground state to singlet excited state presented in Table 2.

Table 2: $S_0 \rightarrow S_1$ energies and oscillator strengths calculated with TDDFT.

**Compound**	**E (eV, nm)**	**f**
**1**	2.85, 436	0.74
**2**	3.11, 398	0.73
**3**	3.30, 376	1.08
**4**	2.92, 424	1.36

VI. Back-isomerization of QC-form using light

A solution of **4** in toluene was prepared in a four-clear-sided cuvette and purged under nitrogen for about an hour. The cuvette was then placed inside a spectrophotometer and irradiated with a 405 nm and 340 nm LED light alternately for 60 s each. The change in absorbance probed at 430 nm was plotted against number of cycles.

VII. References

1. Zehner, R. W.; Parsons B. F.; Hsung, R. P,; Sita, L. R., Tuning the work function of gold with self-assembled monolayers derived from X-[C_6H_4-C≡C-]$_n$$C_6H_4$-SH (n = 0, 1, 2; X = H, F, CH_3, and OCH_3). *Langmuir* 1999, 15, 1121-1127.
2. Tebikachew, B. E.; Li, H. B.; Pirrotta, A.; Borjesson, K.; Solomon, G. C.; Hihath, J.; Moth-Poulsen, K., Effect of Ring Strain on the Charge Transport of a Robust Norbornadiene-Quadricyclane-Based Molecular Photoswitch. *J Phys Chem C* **2017,** *121*, 7094-7100.
3. Hatchard, C. G.; and Parker, C. A., A new sensitive chemical actinometer. Potassium ferrioxalate as a standard chemical actinometer. *Proceedings of the Royal Society of London. Series A, Mathematical and Physical Sciences*, 1956, **235**, 518-536.
4. Stranius, K.; Börjesson, K., Determining the photoisomerization quantum yield of photoswitchable molecules in solution and in the solid state. *Sci. Reports* 2017, *7*, 41145.
5. Demas, J. N. and Crosby, G. A., The measurement of Photoluminescence Quantum Yields. A Review. *J. Phys Chem* 1971, 76, 991-1024.
